# Enhanced Serpent algorithm using Lorenz 96 Chaos-based block key generation and parallel computing for RGB image encryption

**DOI:** 10.7717/peerj-cs.812

**Published:** 2021-12-17

**Authors:** Huwaida T. Elshoush, Banan M. Al-Tayeb, Khalil T. Obeid

**Affiliations:** 1 Department of Computer Science, Faculty of Mathematical Sciences and Informatics, University of Khartoum Khartoum, Sudan; 2 Department of Pure Mathematics, Faculty of Mathematical Sciences and Informatics, University of Khartoum, Khartoum, Sudan

**Keywords:** Serpent, RGB image encryption, Lorenz 96, Chaotic map, Parallel computing

## Abstract

This paper presents a new approach to enhance the security and performance of the Serpent algorithm. The main concepts of this approach is to generate a sub key for each block using Lorenz 96 chaos and then run the process of encryption and decryption in ECB parallel mode. The proposed method has been implemented in Java, openjdk version “11.0.11”; and for the analysis of the tested RGB images, Python 3.6 was used. Comprehensive experiments on widely used metrics demonstrate the effectiveness of the proposed method against differential attacks, brute force attacks and statistical attacks, while achieving superb results compared to related schemes. Moreover, the encryption quality, Shannon entropy, correlation coefficients, histogram analysis and differential analysis all accomplished affirmative results. Furthermore, the reduction in encryption/decryption time was over 61%. Moreover, the proposed method cipher was tested using the Statistical Test Suite (STS) recommended by the NIST and passed them all ensuring the randomness of the cipher output. Thus, the approach demonstrated the potential of the improved Serpent-ECB algorithm with Lorenz 96 chaos-based block key generation (BKG) and gave favorable results. Specifically, compared to existing encryption schemes, it proclaimed its effectiveness.

## Introduction

Nowadays, securing sensitive data is one of the main concerns among researchers/industry professionals. Although Serpent algorithm is secure, it faces limitations such as memory requirement and execution time. The 32 rounds of Serpent affect the performance directly (Odion, 2015). Multifarious image encryption techniques enhancing Serpent algorithm were developed by researchers such as Ahmed, Ali & Hassin (2017), Shah, Haq & Farooq (2018), Yousif (2019), Zagi & Maolood (2020), Ali & Ressan (2016), Kumar & Girdhar (2021), Khan et al. (2012), Ali & Ressan (2016) and Elkamchouchi, Takieldeen & Shawky (2018). In particular, researchers (Elkamchouchi, Takieldeen & Shawky, 2018) applied chaotic maps in improving Serpent. Specifically, Ahmed, Ali & Hassin (2017), Shah, Haq & Farooq (2018), Yousif (2019), Zagi & Maolood (2020) revamp Serpent using S-box based enhancements.

Chaos is considered a prodigious evolution in the field of securing data due to its assorted applications in many areas such as computer science (Al-Hazaimeh et al., 2017). Being unpredictable, random, ergodic and high sensitive to preliminary conditions, make chaotic systems well suited to encryption and secure transmission. In particular, a chaos-based image encryption is the precipitate way for hiding digital images and therefore, is widely used in image encryption schemes (Elkamchouchi, Takieldeen & Shawky, 2018; Al-Hazaimeh et al., 2017; Shah, Haq & Farooq, 2020; Fouda et al., 2014; Kumar & Chandrasekaran, 2009; Kumar et al., 2012; Cavusoglu et al., 2018; Alanazi, 2021; Alkhe, El-Bakry & Fathalla, 2016; Zou et al., 2020). Specifically, researches Al-Hazaimeh et al. (2017) and Zou et al. (2020) utilized Lorenz to enhance the security to resist common attacks in encrypting image. Fouda et al. (2014) utilized linear chaotic map in image block encryption algorithm. Their method can generate large permutation and diffusion keys very fast also having faster and higher security level. In particular, Kumar et al. (2012), which is an improvement to Kumar & Chandrasekaran (2009), propound a fast encryption scheme using Lorenz Attractor. Their work utilizes parallelism without weakening the security.

From another perspective, researchers Tayel, Dawood & Shawky (2018) proffer using two keys to replace the one key used by Serpent. On the other hand, Singh & Singh (2016) suggested applying a different key to every block to speed AES, which gave rise to more unpredictable cipher. Pendli et al. (2016) utilized parallel computing to reduce the execution time of AES. Reckon on their superb results of reduction in time up to 45%, similar algorithms can benefit from parallel implementation (Nagendra & Sekhar, 2014).

In the quest of improving the Serpent performance and security, an enhanced Serpent-256-ECB with Lorenz 96 BKG is proposed. It enhances the security by generating sub keys for each block using the Lorenz 96 chaos-based BKG algorithm, and further run the Serpent in parallel mode to speed it up. Moreover, all block keys can be generated prior to the inception of Serpent, which makes it possible to encrypt each block in parallel and furthermore hide plaintext patterns.

Our contribution can be summarized as follows:

• Speeding up Serpent by splitting the colored image into RGB layer blocks and generating Lorenz 96 chaos-based sub-keys for every block, with parallel implementation.
• Serpent is strengthen, from a security facet, as generating Lorenz 96 chaotic map adds more strength to the algorithm due to the intricate of the key. Moreover, deploying a distinct key for each different block hides plaintext patterns.
• The proposal is tested in encrypting image and proved its efficacy as being fast and secure.
• Furthermore, our proposed method achieved effectual results over the state-of-the-art schemes.

## The Serpent Algorithm

### Serpent encryption process

The Serpent cipher is a key block algorithm that uses a data block of 128 bits and features three key sizes, including 128, 192, or 256 bits. Practically, it is a 32-round system that operates on four 32-bit words, hence the 128-bits block size. Each round applies one of eight 4 × 4 S-boxes 32 times in parallel. It was designed so that all operations can be executed in parallel (Anderson, Biham & Knudsen, 2005; Naeemabadi et al., 2015; Compton, Timm & Van Laven, 2009; Biham, Knudsen & Anderson, 1998). It has three main functions:

#### Initial permutation (IP)

The initial permutation of bits is done by a lookup table to decide which bit to place in which position as defined in the permutation table (Anderson, Biham & Knudsen, 2005; Naeemabadi et al., 2015; Compton, Timm & Van Laven, 2009; Biham, Knudsen & Anderson, 1998).

#### Round function (R)

The algorithm has eight S-boxes (S_*i*_). The round function is performed 32 times on data block B_*i*_. Each round consists of three operations: key mixing XOR, 32 parallel applications of the same 4 × 4 S-box substitution, and linear transformation (LT); except in the last round, the linear transformation is replaced by an additional key mixing XOR operation (Anderson, Biham & Knudsen, 2005; Naeemabadi et al., 2015; Compton, Timm & Van Laven, 2009; Biham, Knudsen & Anderson, 1998; Hari, 2017).

#### Final permutation (FP)

A final permutation of bits is performed to place the bits back into the correct position, as an inverse of the initial permutation. FP can be done *via* lookup table or algorithmically by replacing the bit at position *i* with bit at position *(i* ×* 4) mod 127*, leaving only bits *0* and *127* in place. The output of this final permutation is the final ciphertext of the algorithm (Anderson, Biham & Knudsen, 2005; Naeemabadi et al., 2015; Compton, Timm & Van Laven, 2009; Biham, Knudsen & Anderson, 1998; Hari, 2017).

### Serpent key generation

To perform the 32 rounds of the Serpent algorithm for each block, 33 round keys must be generated from the key provided by the user. Firstly, eight 32 bit keys, *w*_1_ to *w*_8_ will be created by splitting the key provided by the user into 32 bits. After that, the 132 intermediate keys are generated using the following pseudo code (Naeemabadi et al., 2015; Compton, Timm & Van Laven, 2009; Biham, Knudsen & Anderson, 1998):

*For i* = 8* to 131*

*w*_*i*_ = (*w*_*i*−8_⊕*w*_*i*−5_⊕*w*_*i*−3_⊕*w*_*i*−1_⊕*phi*⊕*i*) <  <  < 11

where *phi* is known as golden ratio (hexadecimal 0*x*9*e*3779*b*9), and < <  < is a left rotation

The next step is the generation of 33 round keys from the intermediate keys by running them through the S-boxes, and combining them into 128-bit blocks (Naeemabadi et al., 2015; Compton, Timm & Van Laven, 2009; Biham, Knudsen & Anderson, 1998).

### Serpent decryption process

For the decryption process, the inverse S-boxes, the inverse linear transformation and reverse order of the subkeys are used Anderson, Biham & Knudsen (2005), Naeemabadi et al. (2015), Compton, Timm & Van Laven (2009), Biham, Knudsen & Anderson (1998).

## Chaotic Map

Chaotic map has some good features; speed and low memory requirement. This make it very suitable in encrypting data that needs high memory such as images and audio encryption (Lin et al., 2018; Alwahbani & Elshoush, 2016; Alwahbani & Elshoush, 2018; Audhkhasi, 2009; Kocarev, 2001; Xiao, Liao & Deng, 2005). Other characteristics that make them valuable for cryptography are complex numerical patterns, unpredictably for unknown initial conditions, strong dependence on the initial conditions, based on relatively simple equations, confusion- and diffusion-like properties and determinism (Matthews, 1989; Marco, Martinez & Bruno, 2012; Al-Hazaimeh et al., 2017). Specifically, chaos systems’ property of confusion and diffusion makes them resistant to statistical attacks (Fouda et al., 2014; Kumar & Chandrasekaran, 2009; Kumar et al., 2012).

### Lorenz 96 chaotic map

Lorenz 96 chaotic map is a dynamic system, which is used to generate block keys that generates multiple pseudo numbers based on multiple numbers as input, using Eq. (1) (Lorenz, 1996; Karimi & Paul, 2010):

(1) \begin{eqnarray*} \frac{d{x}_{i}}{dt} =({x}_{i+1}-{x}_{i-2}){x}_{i-1}-{x}_{i}+F.\end{eqnarray*}

This simplest version of the model is described by a periodic system of N (*i* = 1, …, N).

where x_*i*_ is the state of the system, and F is a forcing constant (usually 8). It is assumed that x_−1_ = x_*N*−1_; x_0_ = x_*N*_; x_*N*+1_ = x_1_.

## Related work

A brief survey depicting the analysis and suggested Serpent modifications is scrutinized in this section.

Some researchers (Osvik, 2000; Najafi et al., 2004; Taher, El_Deen & Abo-Elsoud, 2014; Banerjee, 1982; Ivancic, Runje & Kovac, 2001) attempted enhancing the Serpent considering hardware. Recently, the application of chaotic map in image block encryption was evolving. Elkamchouchi, Takieldeen & Shawky (2018) enhance Serpent speed by using chaotic mapping and cycling group instead of S-Box. With these modifications, the number of rounds will become 10 instead of 32 rounds. Another research (Yousif, 2019) works on reducing the number of rounds and time usage utilizing chaotic map. The author propounds dynamic methods for permutation, substitution and key generation based on chaotic maps to get more security, hence achieving best randomness and robustness compared to classical Serpent. Moreover, it has sensitivity to any change in the key. Tayel, Dawood & Shawky (2018) use Elliptic Hybrid Cryptosystem to improve the security of Serpent utilizing two keys instead of one key. Conspicuously, Ali & Ressan (2016) split an image into 512-bit blocks and divide every block into four 128-bit blocks. Next they encrypt the last block using Serpent and expands it into 3 blocks, then runs the new blocks with the other blocks in XOR function. This makes Serpent encryption/decryption process faster than normal.

Noteworthy, working on another aspect, researchers Ahmed, Ali & Hassin (2017) and Shah, Haq & Farooq (2018) ameliorated Serpent by working on its functionality. Specifically, Shah, Haq & Farooq (2018) speed up Serpent by using 4 × 4 S-box and decreasing the number of rounds to 22 instead of 32. These enhancements make the improved Serpent 31 % faster than the traditional but it decreases its security level. In an extended version, Shah, Haq & Farooq (2020) use finite commutative chain ring-based S-boxes that dealt with 8-bit vector instead of 4-bit. This enhances the algebraic complexity of the cipher. Hence, their scheme consumes less time and has a great resistance against statistical and differential attacks.

The above mentioned work clearly manifests that till date, Serpent has not been modified in light of generating Lorenz 96 chaos-based sub-keys for every block, with parallel implementation. Furthermore, from the security facet, Lorenz 96 chaotic map adds more security, as deploying a distinct key for each block hides plaintext patterns and strengthen the algorithm by adding extra intricate to the key. Consequently, this paper proposes running Serpent in parallel in addition to generating different block keys using Lorenz 96 chaotic map. The proposed Serpent is implemented in RGB image encryption to ascertain its efficiency in being fast and secure.

## The Proposed Method

**Figure 1:**
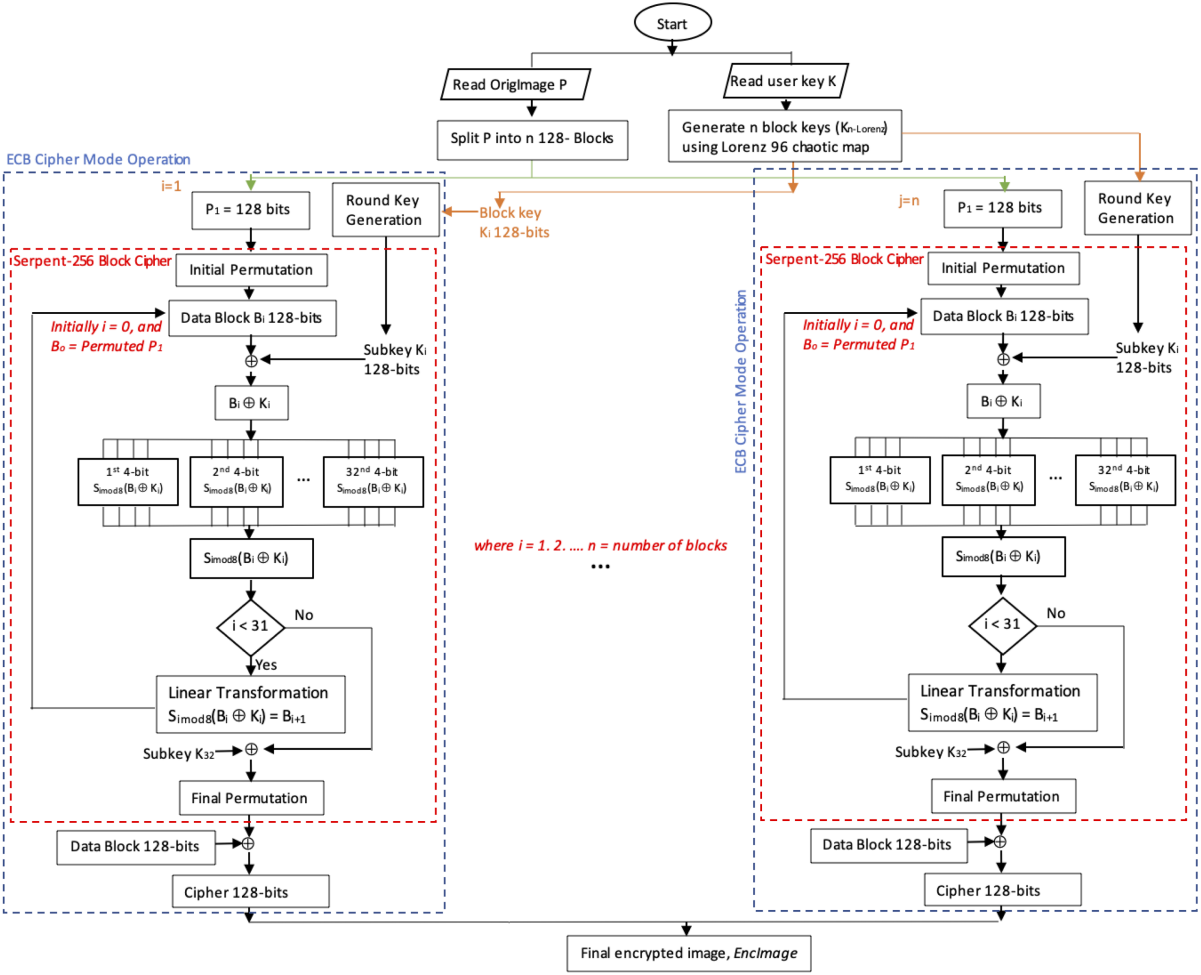
Flowchart depicting the encryption process of the proposed enhanced Serpent-256-ECB with Lorenz 96.

The proposed method, Serpent-256-ECB with Lorenz 96 BKG, enhances the Serpent algorithm by taking advantage of the ECB mode by exploiting parallelism. Lorenz 96 chaotic map was used to generate 256-bit block keys using user input 256-bit key to resolve the data pattern problem in ECB, as outlined in algorithm 1. Note that all of the block keys can be generated prior to the inauguration of Serpent. Figure 1 depicts the encryption process of the proposed method.


 
_______________________ 
 Algorithm 1: Lorenz Key Generator                                               ____ 
    Input: key: Encryption key in array of 32 bytes 
             n: Number of blocks > 1 
    Output:  Kn−Lorenz: an array of n block keys (in bytes) generated using 
                  Lorenz 96 
  1  Function LorenzBlockKeysGeneration(key,n): 
     2   Initialize keylist[n] array to 0           // Initializing array keylist 
3Initialize x[] array to 0                          // Initializing array x 
4x[] ← IntegerOf(key[])             // Convert key byte array into x 
integer array 
5L ← Length(key)                     // L equals length of array key 
6keylist[0] ← ( x[1] - x[L-2] ) × x[L-1] - x[0] + 8     // Key for block 
no.  0 
7keylist[1] ← ( x[2] - x[L-1] ) × x[0] - x[1] + 8  // Key for block no. 
1 
8keylist[L-1] ← ( x[0] - x[L-3] ) × x[L-2] - x[L-1] + 8         // Key for 
block no.  L-1 
9for i = 2 to L-2 do 
 10   keylist[i] ← ( x[i+1] - x[i-2] ) × x[i-1] - x[i] + 8        // Keys for 
block no.  2 to L-2 
11end 
12Kn−Lorenz ← ByteOf(keylist)      // Convert keylist integer array 
into byte array Kn−Lorenz 
13  Return Kn−Lorenz 
14  End Function    


### Encryption using serpent-256-ECB with Lorenz 96 BKG

The proposed encryption method starts with reading the input encryption key from the user. The size of this key must be 256 bits, if it is less than 256 bit then the method adds 0’s to complete the length. Otherwise, if the length is more than 256 bit, the system uses the first 256 bit as key.

Then the proposed method reads the color image and extracts its Red (R), Green (G) and Blue (B) layers. Each layer is then converted into binary and split it into *n* blocks of 128 bits.

Note that if the block number is 1, the image is encrypted using Serpent-256-EBC with the user input encryption key. Otherwise, the method uses Lorenz 96 chaotic map to generate encryption block keys from the user key by calling algorithm 1, *LorenzBlockKeysGeneration*, which has two parameters, namely the user key *key* and the block numbers *n*, where *n* >*1*.

Algorithm 1 returns *K*_*n*−*Lorenz*_ which are *n* Lorenz 96 block keys. These are used to encrypt each RGB layer (block) by calling function *ParSerpentEnc(block[i],keylist[i])* in algorithm 2. This function runs the Serpent encryption in parallel mode with the associated block Lorenz 96 key. The initial permutation is applied to the RGB layers, then the output is XORed with the round keys which are generated using the traditional Serpent round key generation but with Lorenz 96 block key as input instead of the user key. The Serpent operations continue as accustomed.

Finally, the encrypted data is collected and reconverted into bytes that will represent cipher R, G and B layers. Ergo, the encrypted R, G and B layers are combined to produce the encrypted color image, *EncImage*, see Fig. 1. Algorithm 2 outlines the steps.

### Decryption using Serpent-256-ECB with Lorenz 96 BKG

Considering parallelism exploitation, the decryption process is quite similar to the encryption. Nonetheless, the decryption function uses the reverse order of the sub-keys after being generated by Lorenz 96 from the user key, the inverse S-boxes and the inverse linear transformation.

## Experimental Results and Analysis

This section presents series of experiments for evaluating the performance and demonstrating the efficacy of the proposed method in context of time analysis performance, key space analysis, texture analysis, statistical analysis, differential analysis, image quality and the cipher randomness. Among the statistical scrutiny, histogram analysis and adjacent pixels correlation are eminent. NIST Statistical Test Suite was used to measure the randomness. Moreover, the pros and cons together with comparisons with related schemes, were also discussed.

### Preliminaries

The proposed method has been implemented in Java, openjdk version “11.0.11”; and for the analysis of the tested images, Python 3.6 was used. All the experimental results were tested on a laptop with an 8GB RAM, Intel®CoreTM i7-4500U CPU @ 1.80 GHz × 4 processor, and AMD®Hainan/Intel®HD Graphics 4400 (HSW GT2). The OS was a 64-bit Pop 21-OS 21.04.

### Time execution performance

Table 1 presents the encryption and decryption times in seconds of the ten tested images, together with their dimensions and sizes in Kb. Three different sizes of the Lena image, and two of the Baboon image, were tested to compare their different running encryption and decryption times.

**Table 1:** Time analysis of the proposed enhanced serpent.

**Image**	**File dimension**	**File size in Kb**	**Encryption time in seconds**	**Decryption time in seconds**
Lena	512 × 512	473.8	9.5804	9.4524
Lena	440 × 439	338.0	8.056	7.5253
Lena	64 × 64	188.6	4.099	4.219
Baboon	225 × 225	160.1	2.1015	1.8971
Baboon	64 × 64	49.5	0.98	0.988
Cat1	200 × 200	101.1	1.7099	1.5076
Cat2	211 × 185	66.7	1.6411	1.4627
Dog	240 × 210	88.0	2.0665	1.8753
Eye	236 × 225	92.0	2.1178	1.9119
Chameleon	252 × 253	117.1	2.5541	2.3368
Pepper	512 × 512	44.0	10.514	9.5481
Tree	200 × 200	90.2	1.6912	1.5009
Lighthouse	279 × 266	137.1	3.0437	2.7017

### Key space analysis

The key space analysis, which is expressed by the number of probability of breaking the key, is a crucial component for a security cryptosystem. Given *n* as the no. of blocks, the key space is given by:

(2) \begin{eqnarray*}KeySpac{e}_{Serpent-Lorenze}=n\times {2}^{256}.\end{eqnarray*}

It is clear that the key space is large for the proposed method compared to the traditional Serpent. This means a stronger key and hence higher security which proves its efficiency, as its strength depends on the large key space, thus making the brute force attack more difficult.

### Statistical analysis

Statistical analysis has been performed to prove its robustness and resistance against statistical attacks (Shah, Haq & Farooq, 2020; Tayel, Dawood & Shawky, 2018; Pareek, 2012). This is done by testing the Shannon entropy, the distribution of pixels (histograms) of the cipher images, and the correlation coefficient between two adjacent pixels.

#### Shannon entropy

The output encrypted image should be highly random which is evaluated by entropy test. For an ideal scheme, value of entropy should be close to 8. Thus, the values of Table 2 indicate that the proposed method is highly robust against statistical attacks. It is calculated as Shah, Haq & Farooq (2020), Tayel, Dawood & Shawky (2018), Pareek (2012):

(3) \begin{eqnarray*}H(m)=\sum _{i=1}^{{2}^{N}-1}P({m}_{i})lo{g}_{2}[P({m}_{i})].\end{eqnarray*}

**Table 2:** Shannon entropy of the proposed enhanced Serpent-256-ECB with Lorenz 96 BKG.

**Image**	**Shannon entropy**
Lena	7.999705
Baboon	7.998538
Cat1	7.998401
Cat2	7.993051
Dog	7.998788
Eye	7.998824
Chameleon	7.989401
Pepper	7.999732
Tree	7.998420
Lighthouse	7.999253

#### Histogram analysis

**Figure 2:**
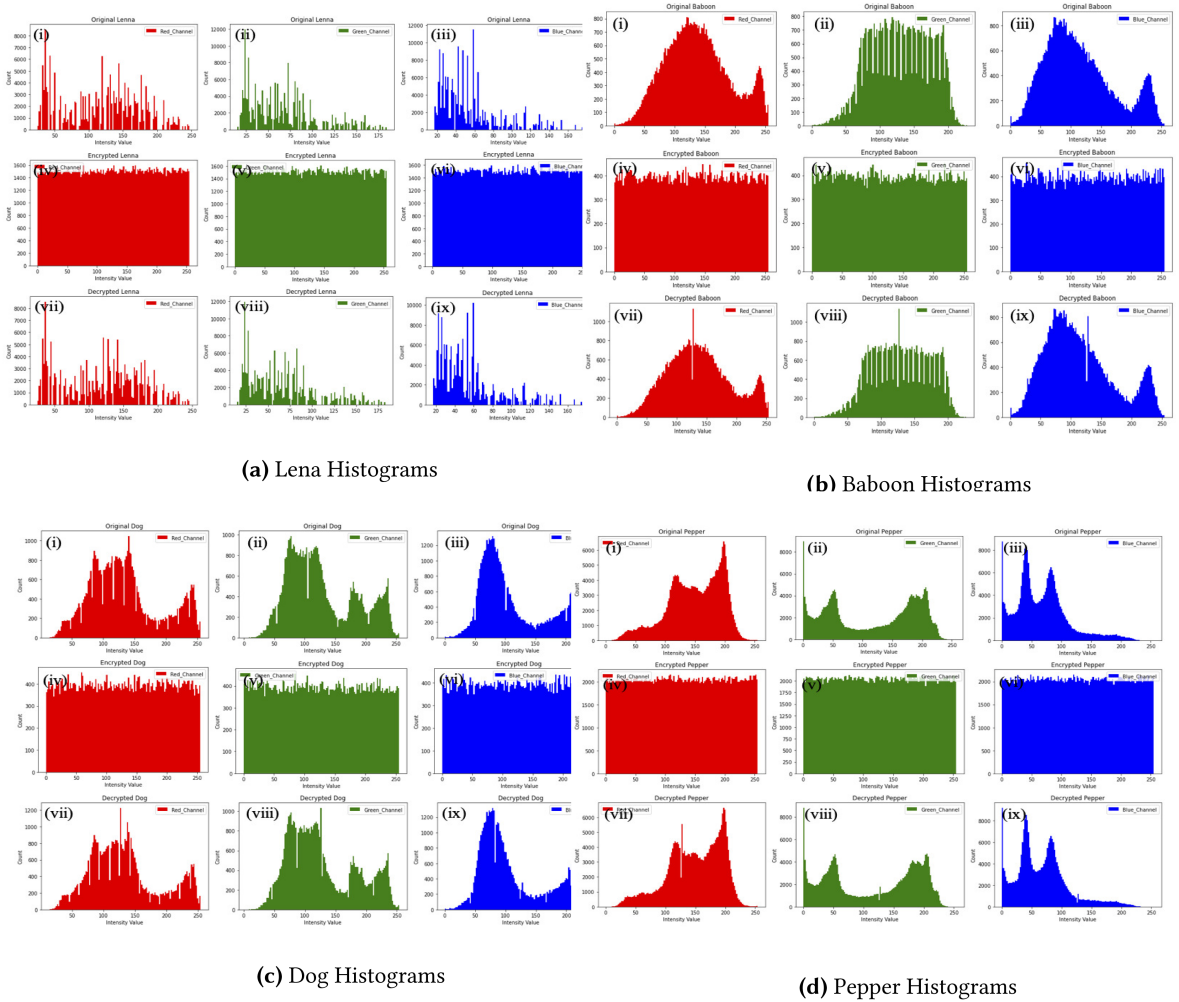
Baboon histograms. (A) Lena histograms, (B) Baboon histograms, (C) Dog histograms, (D) Pepper histograms. (i), (ii), (iii)) shows the histogram of RGB layers of the original images; (iv), (v) and (vi) histograms of the encrypted images, and finally, (vii), (viii) and (ix) depict those of the decrypted Lena, Baboon, Dog and Pepper images respectively.

The bars representation of each byte of an image form what is called image histogram. The sharp edges of these bars represent a weak encryption technique whereas the uniformity of pixels reveals a good encryption scheme that will resist all the statistical attacks (Shah, Haq & Farooq, 2020; Tayel, Dawood & Shawky, 2018; Pareek, 2012). The histograms of four images in Fig. 2 ensures that the proposed method resist statistical analysis attacks. Nevertheless, baboon histograms show insignificant histogram error rate which is reflected by using a scale of 1,000 in the decrypted images compared to 800 in the original RGB.

#### Correlation coefficients

This measures the robustness of a ciphered technique against several attacks. The value 1 is the maximum correlation coefficient, which indicates high correlation between the adjacent pixels. Hence, for a secure ciphered scheme, it should be very low and close to zero (Shah, Haq & Farooq, 2020; Arab, Rostami & Ghavami, 2019). To evaluate the correlation between the two adjacent pixels, the following equations are used:

(4) \begin{eqnarray*}E(x)= \frac{1}{N} \sum _{i}^{N}{x}_{i}\end{eqnarray*}

(5) \begin{eqnarray*}cov(x,y)= \frac{1}{N} \sum _{i}^{N}({x}_{i}-E(x))({Y}_{i}-E(y))\end{eqnarray*}

(6) \begin{eqnarray*}D(x)= \frac{1}{N} \sum _{i}^{N}({x}_{i}-E(x))({y}_{i}-E(y))\end{eqnarray*}

(7) \begin{eqnarray*}r(x,y)= \frac{cov(x,y)}{\sqrt{(D(x))}\sqrt{(D(y))}} , D(x)\not = 0,D(y)\not = 0\end{eqnarray*}

where *x* and *y* denote the values of the two adjacent pixels, and

   *N* is the number of selected adjacent pixels for the correlation calculation.

**Figure 3:**
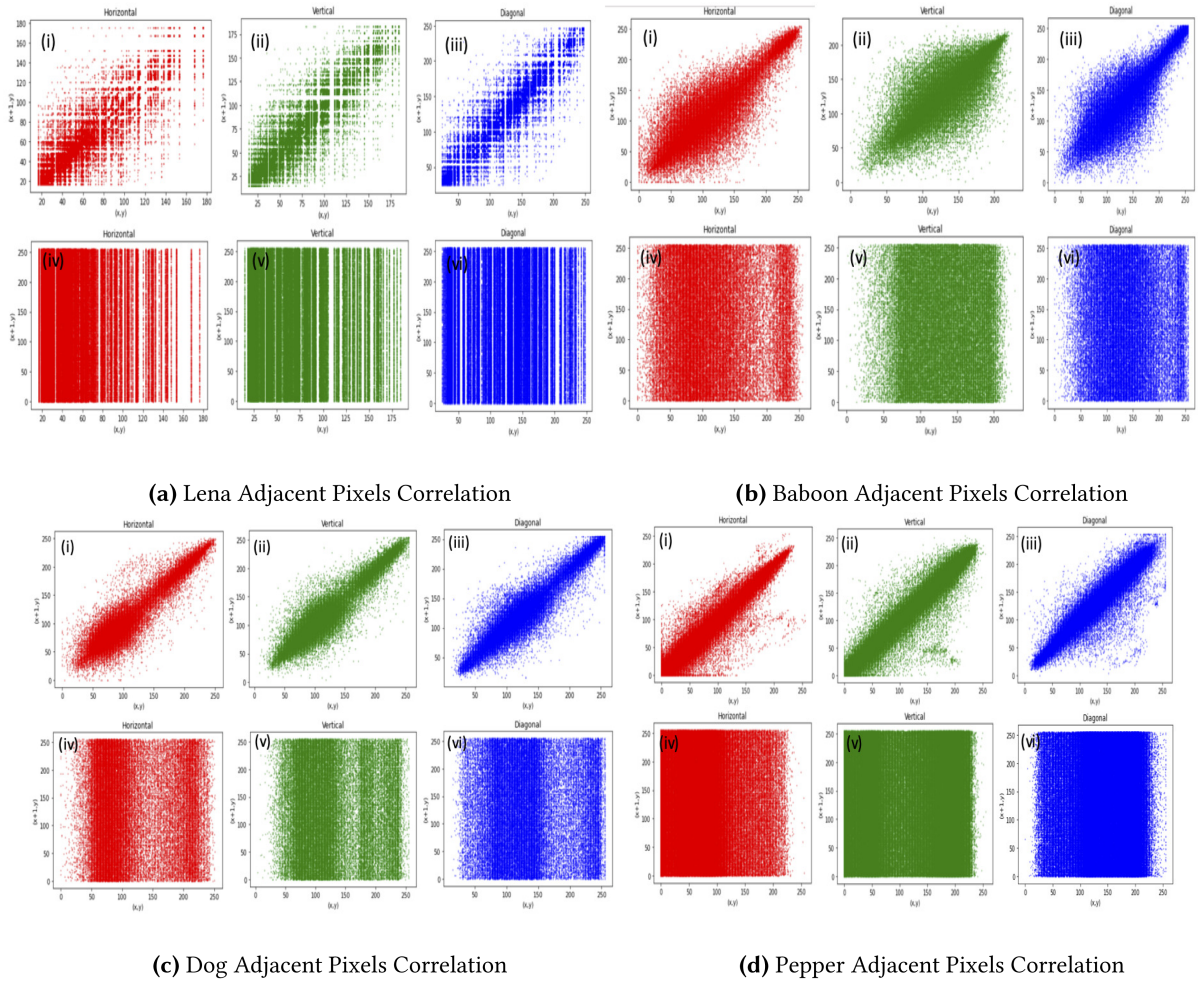
Pepper adjacent pixels correlation. (A) Lena adjacent pixels correlation, (B) Baboon adjacent pixels correlation, (C) Dog adjacent pixels correlation, (D) Pepper adjacent pixels correlation. (i), (ii) and (iii) Represent horizontal, vertical and diagonal correlation of RGB layers of Lena, Baboon, Dog and Pepper original image and (iv), (v) and (vi) depict the horizontal, vertical and diagonal of encrypted Lena, Baboon, Dog and Pepper respectively.

Table 3 proffer the horizontal, vertical, and diagonal correlation for the proposed method for 10 tested images. Clearly, the values of the encrypted images were all close to 0, which affirms the efficiency of the proposed method. Figure 3 depicts the results graphically.

**Table 3:** Horizontal, vertical, and diagonal correlation for the proposed method for 10 images.

**Images**	**Correlation coefficient**					
	**Original & decrypted image**			**Original & encrypted image**		
	**Horizontal**	**Vertical**	**Diagonal**	**Horizontal**	**Vertical**	**Diagonal**
**Lena**	0.999986	0.999985	0.999989	−0.000021	0.001810	−0.000320
**Baboon**	0.999989	0.99998	0.999984	0.005448	0.00898	0.001163
**Cat1**	0.999992	0.999992	0.999991	−0.003417	0.007930	0.009331
**Cat2**	0.999993	0.999991	0.999991	−0.003819	−0.006017	−0.004573
**Dog**	0.999994	0.999990	0.999985	0.002306	−0.003334	0.004846
**Eye**	0.999997	0.999957	0.999985	0.005482	−0.004911	−0.004043
**Chameleon**	0.999999	0.999989	0.999994	0.002088	−0.001019	−0.003147
**Pepper**	0.999989	0.999996	0.9999819	0.000040	0.000926	−0.003259
**Tree**	0.999988	0.9999854	0.999984	−0.010450	0.005445	0.004829
**Lighthouse**	0.999973	0.999981	0.999984	0.009882	−0.000692	−0.006864

### Differential analysis

NPCR is the number of pixels change rate of two encrypted images, their original images are exactly the same except in one pixel. UACI is the average change in intensity between the two encrypted images. They are used to reduce the probability of the differential attack, hence evaluating if the proposed method is vulnerable against the chosen encrypted text attack or an attacker has access to a known plain and encrypted text pair. The terms NPCR and UACI can be calculated by using Eqs. (8) and (9), respectively (Shah, Haq & Farooq, 2020; Tayel, Dawood & Shawky, 2018; Pareek, 2012):

(8) \begin{eqnarray*}NPCR= \frac{\sum _{ij}D(i,j)}{M\times N} \end{eqnarray*}

where, D(i,j) =

\begin{eqnarray*} \left\{ \begin{array}{@{}l@{}} \displaystyle 0, \text{if} {E}_{1}(i,j)={E}_{2}(i,j) \\ \displaystyle 1, \text{if} {E}_{1}(i,j)\not = {E}_{2}(i,j) \\ \displaystyle               \end{array} \right.              \end{eqnarray*}

(9) \begin{eqnarray*}UACI= \frac{1}{M\times N} \sum _{ij}\mid \frac{{E}_{1}(i,j)-{E}_{2}(i,j)}{255} \mid \end{eqnarray*}

Table 4 demonstrates the results of the differential analysis for the ten tested images, which manifest the effectiveness of the proposed method.

**Table 4:** NPCR and UACI of the proposed enhanced Serpent-256-ECB with Lorenz 96 BKG.

**Image**	**NPCR %**	**UACI %**
Lena	99.617761	32.786420
Baboon	99.600988	29.231451
Cat1	99.605833	31.922925
Cat2	99.626831	34.793702
Dog	99.619709	29.990035
Eye	99.583176	31.992903
Chameleon	99.653366	37.082537
Pepper	99.609756	32.156716
Tree	99.610833	30.697134
Lighthouse	99.602950	29.044188

### Encryption quality

Table 5 presents the encryption quality results for each RGB image.

**Table 5:** Encryption quality test values for the proposed method.

**Images**	**MSE**			**PSNR (in dB)**			**SSIM**			**IQI**			**MD**		
	**Red**	**Green**	**Blue**	**Red**	**Green**	**Blue**	**Red**	**Green**	**Blue**	**Red**	**Green**	**Blue**	**Red**	**Green**	**Blue**
**Lena**	11897.1	10735.7	8796.5	7.3764	7.8225	8.6877	0.00692	0.00730	0.00891	0.999998	0.999996	0.99998	246	240	238
**Baboon**	8857.7	7263.2	8376.2	8.6576	9.5195	8.9004	0.0103	0.0111	0.0075	0.99999	0.99998	0.99998	255	237	250
**Cat1**	10345.7	9790.4	9688.4	7.9832	8.2228	8.2683	0.0023	0.0097	0.0106	0.99999	0.99999	0.99999	255	255	255
**Cat2**	11322.6	11445.3	12637.5	7.5913	7.5445	7.1142	0.0074	0.0093	0.0089	0.99998	0.99998	0.99998	255	255	255
**Dog**	8922.2	8565.8	8499.7	8.6261	8.8031	8.8368	0.0087	0.0092	0.0103	0.99999	0.99999	0.99998	255	250	250
**Eye**	14541.2	6859.3	8540.9	6.5048	9.7680	8.8157	0.0055	0.0109	0.0089	0.99999	0.99998	0.99997	255	255	255
**Chameleon**	17154.8	10852.1	11914.8	5.7869	7.7757	7.3699	0.0047	0.0083	0.0080	0.99999	0.99998	0.99999	255	255	255
**Pepper**	11035.1	11075.4	8045.8	7.7030	7.6872	9.0751	0.0076	0.0078	0.0089	0.99999	0.99998	0.99998	251	255	255
**Tree**	9908.8	8806.4	8699.0	8.1706	8.6828	8.7361	0.0049	0.0083	0.0118	0.99997	0.99997	0.99997	237	237	235
**Lighthouse**	8374.8	7683.1	8024.7	8.9011	9.2754	9.0865	0.0122	0.0110	0.0074	0.99999	0.99999	0.99999	255	251	255

#### Mean square error MSE)

MSE is the cumulative squared difference between the original image P(x,y) and encrypted image C(x,y). A greater value for MSE is perceived as better first-rate (Shah, Haq & Farooq, 2020; Tayel, Dawood & Shawky, 2018; Pareek, 2012).

(10) \begin{eqnarray*}MSE= \frac{1}{MN} \sum _{y=1}^{M}\sum _{x=1}^{N}[P(x,y)-C(x,y)]^{2}.\end{eqnarray*}

#### Peak signal to noise ratio (PSNR)

PSNR is the ratio of maximum intensity value (MAX) of the original image, which is 255, to that of the encrypted image. For a good crypto-system, a low value of PSNR is required, which depicts a significant difference between plain and encrypted images (Shah, Haq & Farooq, 2020; Tayel, Dawood & Shawky, 2018; Pareek, 2012). The effectiveness of the proposed technique is evaluated using PSNR in decibel, using Eq. (11), thus indicating a higher quality of encryption, see Table 5.

(11) \begin{eqnarray*}PSNR=10{\log \nolimits }_{10} \frac{MA{X}^{2}}{MSE} .\end{eqnarray*}

#### Structural similarity index measure (SSIM)

For n × n size of image having X and Y parts, the SSIM is calculated as Shah, Haq & Farooq (2020); Tayel, Dawood & Shawky (2018); Pareek (2012):

(12) \begin{eqnarray*}SSIM(c,s)= \frac{(2{\mu }_{X}2{\mu }_{Y}+v1)(2{\sigma }_{XY}+v2)}{({{\mu }_{X}}^{2}+{{\mu }_{Y}}^{2}+v1)({{\sigma }_{X}}^{2}+{{\sigma }_{Y}}^{2}+v2)} \end{eqnarray*}

where *μ*_*X*_ = average of X, *μ*_*Y*_ = average of Y, *σ*^2^*X* = variance of X, *σ*^2^*Y* = variance of Y, *σ*_*XY*_ = covariance of X and Y, c_1_ =(k_1_ L)^2^, and c_2_ =(k_2_ L)^2^ (variables to stabilize the division with small value of denominator), and L = vibrant range of the pixel values, (k_1_, k_2_) = (0.01, 0.03) by default.

#### Image quality Index (IQI)

IQI is used to figure out any change in the image correlation, luminance, and contrast. Its values range from −1 to 1 (Shah, Haq & Farooq, 2020; Tayel, Dawood & Shawky, 2018; Pareek, 2012).

#### Maximum difference (MD)

MD is the maximum difference between pixels of two images, 0 means no difference (Shah, Haq & Farooq, 2020; Tayel, Dawood & Shawky, 2018; Pareek, 2012).

(13) \begin{eqnarray*}MD=MAX(P(x,y)-C(x,y))\end{eqnarray*}

### The NIST statistical test suite (STS)

**Figure 4:**
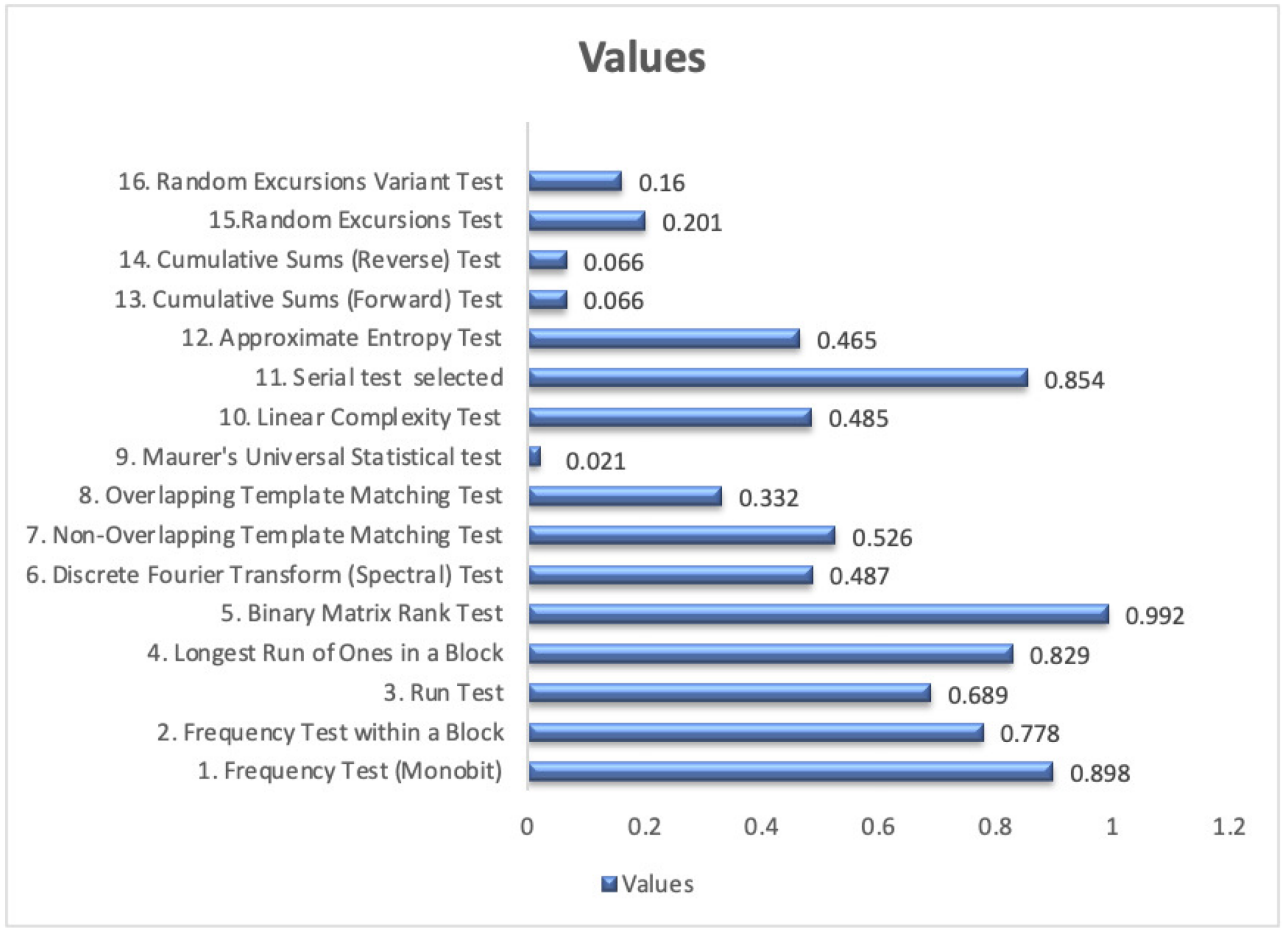
The NIST Statistical Test Suite (STS) for an input of zeros and ones.

The Statistical Test Suite (STS) recommended by the NIST has 16 different tests. The cipher passes any particular test if the calculated probability *P*-value is in the range 0.01 ≤P ≤1 (Bassham et al., 2010; Sỳs & Řìha, 2014; Marton & Suciu, 2015). The tests were repeated several times and all different inputs passed all the NIST tests, as depicted in Fig. 4, which advocate that it is statistically indistinguishable from a random output.

### Advantages and disadvantages of the proposed method

The results of testing the proposed method yields the following pros and cons:

#### Advantages

1. This method enhanced the performance by speeding up Serpent. This is achieved by splitting the colored image into RGB layer blocks and generating Lorenz 96 chaos-based block sub-keys, then running it in parallel mode.
2. All of the block keys can be generated prior to the inception of Serpent.
3. Using EBC mode and running Serpent RGB layers in parallel mode hide plaintext patterns.
4. The generation of block sub-keys by Lorenz 96 enhances the security as chaos systems’ property of confusion and diffusion makes them resistant to statistical attacks.
5. Chaotic map suits encrypting data that needs high memory such as images encryption due to its speed and low memory requirement.
6. Complex numerical patterns and unpredictably for unknown initial conditions makes Lorenz 96 chaotic map ameliorate the Serpent’s security.

#### Disadvantages

1. Noise attacks may affect the image quality.
2. Cropping will possibly affect the retrieval of the original image.

### Comparison with related schemes

In order to highlight the overall potential of the proposed method, it is juxtaposed with related schemes and compared in terms of time execution, key space, statistical analysis, NPCR and UACI, and encryption quality.

#### Comparing the time execution performance

Table 6 shows the time taken by the proposed method for encrypting and decrypting the RGB Baboon and Lena image of different sizes. The execution of the proposed method, compared to traditional Serpent, shows a reduction 61.25 % in encryption time and 61.37 % in decryption time for Baboon. Lena encryption time was reduced by 67.55 % and decryption time by 71.35 %.

**Table 6:** Time execution performance efficiency comparison.

**Image (128-bit block size)**	**Method**	**Encryption time in seconds**	**Decryption time in seconds**	**Reduction in encryption time**	**Reduction in decryption time**
Baboon	Serpent-256-ECB	0.98	0.988	61.25%	61.37%
(50640 bytes)	Traditional Serpent	1.60	1.61		
Lena	Serpent-256-ECB	4.099	4.219	67.55%	71.35%
(193168 bytes)	Traditional Serpent	6.068	5.913		

#### Comparison of key space analysis

Table 7 shows key space analysis for different encryption schemes. Our method outperforms the related schemes especially for large images (more than one block). Undoubtedly, this ascertains that the proposed encryption method is robust and resistant against brute force attack.

**Table 7:** Comparison of key space for the proposed method and related encryption schemes.

	**Proposed method**	**Tayel, Dawood & Shawky (2018)**	**Yousif (2019)**	**Shah, Haq & Farooq (2020)**	**Zou et al. (2020)**	**Traditional serpent**
**Key space**	**n ×2^256^**	2^256^	10^112^	2^264^	2^232^	2^256^
	*(n = no. of blocks)*					

#### Comparison of shannon entropy values

Considering Lena, Baboon and Pepper images, Table 8 presents a comparison of some related schemes Shannon Entropy values and the proposed method. In particular (Kumar et al., 2012) work uses chaos theory and parallelism achieving 7.9934 entropy whereas ours has a value of 7.999705 for Lena image. For instance, the Serpent improvement of Shah, Haq & Farooq (2020) attained a value of 7.9992 for the same image. Evidently, our proposed method surpasses the related schemes.

**Table 8:** Comparison of shannon entropy values between the proposed enhanced serpent and existing image encryption schemes.

**Images**	**Image encryption algorithm**	**Reference**	**Shannon entropy**
**Lena**	**Serpent-based**	**Proposed**	**7.999705**
		**Ali & Ressan (2016)**	7.5975
		**Tayel, Dawood & Shawky (2018)**	7.2341
		**Shah, Haq & Farooq (2020)**	7.9992
		**Traditional Serpent**	7.2341
	**Others**	**Kumar et al. (2012)**	7.9934
		**Annadurai, Manoj & Jathanna (2017)**	7.9997
		**Cavusoglu et al. (2018)**	7.9577
		**Zou et al. (2020)**	7.9991
		**AES**	7.8693
**Baboon**	**Serpent-based**	**Proposed**	**7.998538**
		**Ali & Ressan (2016)**	7.6310
		Tayel, Dawood & Shawky (2018)	7.2216
		**Traditional Serpent**	7.2216
	**Others**	Zou et al. (2020)	7.9991
**Pepper**	**Serpent-based**	**Proposed**	**7.999732**
	**Others**	**AES**	7.8734
		Zou et al. (2020)	7.9991
		Alanazi (2021)	7.999049

#### Comparative analysis of the adjacent pixels correlation

Using Lena, Baboon and Pepper images, Table 9 presents a comparative analysis of the correlation coefficient of the proposed method with some related schemes, standard AES and the traditional Serpent. Compared to Serpent enhancement of Shah, Haq & Farooq (2020) and the chaotic-based with parallelism scheme of Kumar et al. (2012), our method achieved far better values of correlation coefficient which ensures the resistance of the proposed method to statistical attacks. Referring to Table 9, blatantly the proposed method coefficients are very low in the encrypted image and approaching zero, hence excelling the related image encryption schemes.

**Table 9:** Comparison of the proposed method’s horizontal, vertical, and diagonal correlation with existing image encryption schemes.

**Images**	**Image encryption algorithm**	**Reference**	**Correlation coefficient between**					
			**Original & decrypted image**			**Original & encrypted image**		
			**Horizontal**	**Vertical**	**Diagonal**	**Horizontal**	**Vertical**	**Diagonal**
**Lena**	**Serpent-based**	**Proposed**	**0.999986**	**0.999985**	**0.999989**	**−0.00002**	**0.001810**	** −0.00032**
		Shah, Haq & Farooq (2020)	0.9371	0.9464	0.8391	0.007	−0.0009	0.0088
		Elkamchouchi, Takieldeen & Shawky (2018)	–	–	–	0.042	−0.043	0.074
		Tayel, Dawood & Shawky (2018)	0.9752	0.9859	0.9623	0.0080	−0.00001	0.0146
		Ali & Ressan (2016)	–	–	–	0.01252	0.01594	0.01348
		**Traditional Serpent**	–	–	–	−0.084	0.125	−0.09
	**Others**	Zou et al. (2020)	0.9765	0.9606	0.9356	0.0032	−0.0004	0.0059
		Annadurai, Manoj & Jathanna (2017)	0.9503	0.9655	0.9373	−0.00097	0.000902	0.00225
		Alkhe, El-Bakry & Fathalla (2016)	0.9900	0.9858	–	0.0037	0.0045	–
		Kumar et al. (2012)	0.9681	0.9821	0.9819	0.0219	0.0230	0.0208
		**AES**	–	–	–	0.07	−0.064	0.121
**Baboon**	**Serpent-based**	**Proposed**	**0.999989**	**0.99998**	**0.999984**	**0.005448**	**0.00898**	**0.001163**
		**Shah, Haq & Farooq (2020)**	0.9229	0.7461	0.8431	0.0062	0.0008	0.0046
		Tayel, Dawood & Shawky (2018)	0.8631	0.7675	0.7335	0.00028	0.0201	0.0042
		Ali & Ressan (2016)	–	–	–	0.01521	0.0110	0.0182
**Pepper**	**Serpent-based**	**Proposed**	**0.999989**	**0.999996**	**0.999982**	**0.00004**	**0.000926**	**-0.00326**
		Tayel, Dawood & Shawky (2018)	0.9800	0.9825	0.9703	0.00048	−0.0387	−0.0062

#### Differential analysis comparison

Considering NPCR and UACI, the juxtaposition of the results of the proposed method and related schemes is displayed in Table 10. The optimal NPCR value should be near to 99.6094%, while our method’s value is 99.6178, which is far better than Shah, Haq & Farooq (2020) and Tayel, Dawood & Shawky (2018) proclaiming its effectual results and resistance to differential attacks.

**Table 10:** Comparison of NPCR and UACI test values between the proposed enhanced serpent and existing image encryption schemes.

**Images**	**Image encryption algorithm**	**Reference**	**NPCR %**	**UACI %**
**Lena**	**Serpent-based**	**Proposed**	**99.6178**	**32.7864**
		Shah, Haq & Farooq (2020)	99.6206	30.53
		Tayel, Dawood & Shawky (2018)	99.4190	33.3553
	**Others**	Zou et al. (2020)	99.6246	33.5118
		Cavusoglu et al. (2018)	99.6198	31.58
		Annadurai, Manoj & Jathanna (2017)	99.49	33.4475
**Baboon**	**Serpent-based**	**Proposed**	**99.6010**	**29.2315**
		Yousif (2019)	99.8022	33.3382
		Tayel, Dawood & Shawky (2018)	99.2149	33.2084
	**Others**	Zou et al. (2020)	99.5885	33.4590
**Pepper**	**Serpent-based**	**Proposed**	**99.6098**	**32.1567**
	**Others**	Zou et al. (2020)	99.6048	33.3828

#### Encryption quality comparison

Table 11 demonstrates the encryption quality test values compared to Shah et al. Shah, Haq & Farooq (2020) image encryption scheme. Concerning the PSNR and SSIM, the lower values are better, conversely MSE values should be high. Ergo, our proposed method were superior to Shah, Haq & Farooq (2020) in all values achieved.

**Table 11:** Comparison of encryption quality test values for the proposed method and Shah, Haq & Farooq (2020) for the Lena image.

**Quality measure**	**Proposed method**			**Shah, Haq & Farooq (2020)**		
	**Red**	**Green**	**Blue**	**Red**	**Green**	**Blue**
**MSE**	11897.1	10735.7	8796.5	10630	9155.2	7196.8
**PSNR**	7.3764	7.8225	8.6877	7.8653	8.5141	9.5593
**SSIM**	0.00692	0.00730	0.00891	0.0103	0.0092	0.0096
**MD**	246	240	238	255	247	211

## Conclusion and Future Work

Enhancing the security and performance of the Serpent algorithm is proposed. The Serpent algorithm is run in parallel ECB mode and a key is generated for every block using Lorenz96 Chaotic map. Based on the experimental results, it was concluded that the parallel implementation of Serpent algorithm is an appropriate method when the performance is the main concern. The proposed method was implemented on the image. The image encryption implementation on ten tested colored images showed high reductions in encryption time of over 61% and 71% for decryption time compared to traditional Serpent. A very large key space provides higher security and assured the strength against brute force attacks. Entropy analysis of the encrypted images gives a value close to the theoretical value 8, and moreover better results when compared with prevailing methods. Concerning the statistical attacks, the analysis of the adjacent pixels correlation and the histogram analysis of the proposed method were scrutinized. It is evident from the close to zero correlation coefficients of the encrypted RGB images that the enhanced Serpent achieved great results and surpass existing schemes. The histogram analysis shows a uniform distribution of pixel intensities, also confirming the effectiveness of the proposed method. The differential analysis for the proposed Serpent-ECB with Lorenz96 shows that NPCR and UACI exceed the expected values, so even a slight change in the original image results in a significant change in the encrypted image. Additionally, it performed better than related schemes NPCR and UACI. In the encryption quality test, the resulted values for the proposed method reflects that the encrypted data is not similar to the original one. Furthermore, the proposed cipher passed all the Statistical Test Suite (STS) recommended by the NIST which ensures the randomness of the cipher output. Thus, the outcome proved the effectiveness of the proposed approach and the Serpent algorithm’s performance and security are significantly improved. Furthermore, it surpasses prevailing encryption schemes. Moreover, the results show excellent potential for practical encryption applications, specifically real time image encryption.

For future work, we recommend to test the proposed method against noise attacks, cropping and rotating the original image. Furthermore, running the Serpent with Lorenz 96 block key generation in CTR cipher mode should be tested, as it is expected to achieve better results.

## Supplemental Information

10.7717/peerj-cs.812/supp-1 Supplemental Information 1 Analysis and the Implementation Results of the Ten Tested Images Includes the numerical and graphical results.

10.7717/peerj-cs.812/supp-2 Supplemental Information 2 Encryption and Decryption Time Analysis The encryption and decryption running times for the ten tested images.

## Additional Information and Declarations

### Data Availability

The following information was supplied regarding data availability: The data is available at GitHub: https://github.com/khalileibad/Serpent_MSC?files=1.
