# Supplementary material for: Enhanced Serpent algorithm using Lorenz 96 Chaos-based block key generation and parallel computing for RGB image encryption
Source: PeerJ Comput Sci. 2021 Dec 17;7:e812. doi: 10.7717/peerj-cs.812 (PMC8725658; doi:10.7717/peerj-cs.812)
Supplement: Supplemental Information 2 — The encryption and decryption running times for the ten tested images. [file peerj-cs-07-812-s002.pdf]

Running times

	Avg Enc time	Avg Dec time	File Size
Baboon	2101.5	1897.1	160.1kB
Cat1	1709.9	1507.6	101.1kB
Cat2	1641.1	1462.7	66.7kB
Chameleon	2554.1	2336.8	117.1kB
Dog	2066.5	1875.3	88.0kB
Eye	2117.8	1911.9	92.0kB
Lenna	9580.4	9452.4	473.8kB
Lighthouse	3043.7	2701.7	137.1kB
Pepper	10514	9548.1	44.0kB
Tree	1691.2	1500.9	90.2kB
Lennaorig	8056	7525.3	338.0kB

Memory: 8GB
Processor: Intel® Core™ i7-4500U CPU @ 1.80GHz × 4
Graphics: AMD® Hainan / Intel® HD Graphics 4400 (HSW GT2)

OS name: Pop!_OS 21.04
OS type: 64-bit

Serpent Language: Java, openjdk version "11.0.11"
Analysis Language: Python 3.6

File Dims in bits
225x225
200x200
211x185
252x253
240x210
236x225
512x512
279x266
512x512
200x200
440x439
