# Supplementary material for: Enhanced Serpent algorithm using Lorenz 96 Chaos-based block key generation and parallel computing for RGB image encryption
Source: PeerJ Comput Sci. 2021 Dec 17;7:e812. doi: 10.7717/peerj-cs.812 (PMC8725658; doi:10.7717/peerj-cs.812)

In [1]:

```python
# Based on: https://github.com/ankit41/CollegeProject
# 1. Import the necessary packages
from PIL import ImageChops,Image
import math, operator
import numpy as np
from skimage import io
from skimage import metrics
from skimage import measure
import imutils
import cv2
import matplotlib.pyplot as plt
from sewar.full_ref import uqi

"""
    It has a NPCR(Number of pixel changed rate).It compares the pixel values of
    The resultant value is returned in percentage.If the value is more than 99%

    It consist of three modules rateofchange(),sumofpixel(),npcrv().
"""

"""
    This method compares two image.If the pixel value is same then 0 is stored i
"""
def rateofchange(height,width,pixel1,pixel2,matrix,i):

    for y in range(0,height):
        for x in range(0,width):
            #print(x,y)
            if pixel1[x,y][i] == pixel2[x,y][i]:
                matrix[x,y]=0
            else:
                matrix[x,y]=1
    return matrix
"""
    sum of the values of 1 stored in matrix is calculated
"""
def sumofpixel(height,width,pixel1,pixel2,ematrix,i):
    matrix=rateofchange(height,width,pixel1,pixel2,ematrix,i)
    psum=0
    for y in range(0,height):
        for x in range(0,width):
            psum=matrix[x,y]+psum
    return psum

"""
Finally the above two module is called to calculate the values
"""
def npcrv(c1,c2):
    width, height = c1.size
    pixel1 = c1.load()
    pixel2 = c2.load()
    ematrix = np.empty([width, height])
    per=(((sumofpixel(height,width,pixel1,pixel2,ematrix,0)/(height*width))*100)
    return per

"""
It calculates the difference between two images.This is just thee coding of a ma
This function can called by passing two image object as parameters and then deci
"""

def msecalc(imageA, imageB):
    # the 'Mean Squared Error' between the two images is the
    # sum of the squared difference between the two images;
    # NOTE: the two images must have the same dimension
    err = np.sum((imageA.astype("float") - imageB.astype("float")) ** 2)
    err /= float(imageA.shape[0] * imageA.shape[1])

    # return the MSE, the lower the error, the more "similar"
    # the two images are
    return err

# Same as NPCR both are used for sensitivity analysis two encrypted image is req

"""
Unified average avereage inensity changed rate
this code calculates average intensity change rate of pixels
"""
def uaciv(image1,image2):
    pixel1=image1.load()
    pixel2=image2.load()
    width,height=image1.size
    value=0.0
    for y in range(0,height):
        for x in range(0,width):
            value=(abs(pixel1[x,y][0]-pixel2[x,y][0])/255)+value

    value2=0.0
    for y in range(0,height):
        for x in range(0,width):
            value2=(abs(pixel1[x,y][1]-pixel2[x,y][1])/255)+value2

    value3=0.0
    for y in range(0,height):
        for x in range(0,width):
            value3=(abs(pixel1[x,y][2]-pixel2[x,y][2])/255)+value3

    value=(value/(width*height))*100
    value2=(value2/(width*height))*100
    value3=(value3/(width*height))*100
    value4=(value+value2+value3)/3
    return (value4, value,value2,value2)

def mdv(imageA, imageB):
    pixel1=imageA.load()
    pixel2=imageB.load()
    width, height = imageA.size
    maxR = abs(pixel1[0,0][0]-pixel2[0,0][0])
    maxG = abs(pixel1[0,0][1]-pixel2[0,0][1])
    maxB = abs(pixel1[0,0][2]-pixel2[0,0][2])
    for y in range(0,height):
        for x in range(0,width):
            maxR = max(abs(pixel1[x,y][0]-pixel2[x,y][0]),maxR)
            maxG = max(abs(pixel1[x,y][1]-pixel2[x,y][1]),maxG)
            maxB = max(abs(pixel1[x,y][2]-pixel2[x,y][2]),maxB)

    return (maxR,maxG,maxB)
```

In [2]:

```python
# 2. Load the two input images

imA = io.imread("~/Desktop/Paper_Files/Images_Analysis/img/Lenna.png")
imB = io.imread("~/Desktop/Paper_Files/Images_Analysis/img/Lenna_E.png")
imC = io.imread("~/Desktop/Paper_Files/Images_Analysis/img/Lenna_D.png")


im1 = Image.open("/Users/banan/Desktop/Paper_Files/Images_Analysis/img/Lenna.png
im2 = Image.open("/Users/banan/Desktop/Paper_Files/Images_Analysis/img/Lenna_E.p
im3 = Image.open("/Users/banan/Desktop/Paper_Files/Images_Analysis/img/Lenna_D.p

img1 = cv2.imread("/Users/banan/Desktop/Paper_Files/Images_Analysis/img/Lenna.pn
img2 = cv2.imread("/Users/banan/Desktop/Paper_Files/Images_Analysis/img/Lenna_E.
img3 = cv2.imread("/Users/banan/Desktop/Paper_Files/Images_Analysis/img/Lenna_D.

# 4. Compute the Structural Similarity Index (SSIM) between the two
#    images, ensuring that the difference image is returned
(scorer,scoreg,scoreb) = metrics.structural_similarity(img1[:,:,0], img2[:,:,0],

# 5. Compute Shanon Entropy for encrypted images
entropy  = measure.shannon_entropy(img2)

#6 Compute NPCR, UACI, MSE and PSNR between origninal and encrypted

npcr  = npcrv(im1, im2)
uaci = uaciv(im1, im2)
mser,mseg,mseb  = msecalc(img1[:,:,0], img2[:,:,0]),msecalc(img1[:,:,1], img2[:,
psnrr,psnrg,psnrb = cv2.PSNR(img1[:,:,0], img2[:,:,0]),cv2.PSNR(img1[:,:,1], img


#7.Compute MSE and IQI between the original and decrypted
msedr,msedg,msedb  = msecalc(img1[:,:,0], img3[:,:,0]),msecalc(img1[:,:,1], img3
iqir,iqig,iqib = uqi(img1[:,:,0],img3[:,:,0]),uqi(img1[:,:,1],img3[:,:,1]), uqi(

#8. Compute MD
mdr, mdg, mdb = mdv(im1,im2)
cfr, cfg, cfb = np.corrcoef(img1[:,:,0].flat, img3[:,:,0].flat)[0][1], np.corrco
cfer, cfeg, cfeb = np.corrcoef(img1[:,:,0].flat, img2[:,:,0].flat)[0][1], np.cor

#9. Printing
print("SSIM Red: {}".format(scorer[0])+" Green: {}".format(scoreg[0])+" Blue: {}
print("Shannon Entropy: {}".format(entropy))
print("NPCR: {}".format(npcr))
print("UACI: {}".format(uaci))
print("MSE between original and encrypted Red: {}".format(mser)+" Green: {}".for
print("MSE between original and decrypted: Red: {}".format(msedr)+" Green: {}".f
print("PSNR Red: {}".format(psnrr)+" PSNR Green: {}".format(psnrg)+" PSNR Blue:
print("MD Red: {}".format(mdr)+" MD Green: {}".format(mdg)+" MD Blue: {}".format
print("IQI Red: {}".format(iqir)+" Green: {}".format(iqig)+" Blue: {}".format(iq
print("CF between original and encrypted Red: {}".format(cfer)+" Green: {}".form
print("CF btween original and decrypted Red: {}".format(cfr)+" Green: {}".format

# Histograms
_ = plt.hist(imA[:, :, 0].ravel(), bins = 128, color = 'Red', alpha = 1)
_ = plt.xlabel('Intensity Value')
_ = plt.ylabel('Count')
_ = plt.legend(['Red_Channel'])
_ = plt.title('Original Lenna')

plt.show()

_ = plt.hist(imA[:, :, 1].ravel(), bins = 128, color = 'Green', alpha = 1)
_ = plt.xlabel('Intensity Value')
_ = plt.ylabel('Count')
_ = plt.legend(['Green_Channel'])
_ = plt.title('Original Lenna')

plt.show()

_ = plt.hist(imA[:, :, 2].ravel(), bins = 128, color = 'Blue', alpha = 1)
_ = plt.xlabel('Intensity Value')
_ = plt.ylabel('Count')
_ = plt.legend(['Blue_Channel'])
_ = plt.title('Original Lenna')

plt.show()

_ = plt.hist(imB[:, :, 0].ravel(), bins = 128, color = 'red', alpha = 1)
_ = plt.xlabel('Intensity Value')
_ = plt.ylabel('Count')
_ = plt.legend(['Red_Channel'])
_ = plt.title('Encrypted Lenna')

plt.show()

_ = plt.hist(imB[:, :, 1].ravel(), bins = 128, color = 'Green', alpha = 1)
_ = plt.xlabel('Intensity Value')
_ = plt.ylabel('Count')
_ = plt.legend(['Green_Channel'])
_ = plt.title('Encrypted Lenna')

plt.show()

_ = plt.hist(imB[:, :, 2].ravel(), bins = 128, color = 'blue', alpha = 1)
_ = plt.xlabel('Intensity Value')
_ = plt.ylabel('Count')
_ = plt.legend(['Blue_Channel'])
_ = plt.title('Encrypted Lenna')

plt.show()

_ = plt.hist(imC[:, :, 0].ravel(), bins = 128, color = 'red', alpha = 1)
_ = plt.xlabel('Intensity Value')
_ = plt.ylabel('Count')
_ = plt.legend(['Red_Channel'])
_ = plt.title('Decrypted Lenna')

plt.show()

_ = plt.hist(imC[:, :, 1].ravel(), bins = 128, color = 'Green', alpha = 1)
_ = plt.xlabel('Intensity Value')
_ = plt.ylabel('Count')
_ = plt.legend(['Green_Channel'])
_ = plt.title('Decrypted Lenna')

plt.show()

_ = plt.hist(imC[:, :, 2].ravel(), bins = 128, color = 'blue', alpha = 1)
_ = plt.xlabel('Intensity Value')
_ = plt.ylabel('Count')
_ = plt.legend(['Blue_Channel'])
_ = plt.title('Decrypted Lenna')

plt.show()

width, height = im1.size
_ = plt.scatter(img1[1:, :, 0].ravel(),img3[:height-1, :, 0].ravel(), s=1, c = '
_ = plt.xlabel('(x,y)')
_ = plt.ylabel('(x+1,y)')
_ = plt.title('Horizontal')

plt.show()

_ = plt.scatter(img1[1:, :, 1].ravel(),img3[:height-1, :, 1].ravel(), s=1, c = '
_ = plt.xlabel('(x,y)')
_ = plt.ylabel('(x+1,y)')
_ = plt.title('Vertical')
plt.show()

_ = plt.scatter(img1[1:, :, 2].ravel(),img3[:height-1, :, 2].ravel(), s=1, c = '
_ = plt.xlabel('(x,y)')
_ = plt.ylabel('(x+1,y)')
_ = plt.title('Diagonal')
plt.show()

_ = plt.scatter(img1[1:, :, 0].ravel(),img2[:height-1, :, 0].ravel(), s=1, c = '
_ = plt.xlabel('(x,y)')
_ = plt.ylabel('(x+1,y)')
_ = plt.title('Horizontal')
plt.show()

_ = plt.scatter(img1[1:, :, 1].ravel(),img2[:height-1, :, 1].ravel(), s=1, c = '
_ = plt.xlabel('(x,y)')
_ = plt.ylabel('(x+1,y)')
_ = plt.title('Vertical')
plt.show()

_ = plt.scatter(img1[1:, :, 2].ravel(),img2[:height-1, :, 2].ravel(), s=1, c = '
_ = plt.xlabel('(x,y)')
_ = plt.ylabel('(x+1,y)')
_ = plt.title('Diagonal')
plt.show()
```

```
SSIM Red: 0.006916173474911643 Green: 0.007303277985947986 Blue: 0.0089062787075
99267
Shannon Entropy: 7.999705040642481
NPCR: 99.61776075101814
UACI: (32.78642041479876, 30.21995379225675, 33.17765217498362, 33.1776521749836
2)
MSE between original and encrypted Red: 11897.103453095879 Green: 10735.72122592
6693 Blue: 8796.548829985504
MSE between original and decrypted: Red: 0.043958376475460754 Green: 0.091644232
76040588 Blue: 0.09164423276040588
PSNR Red: 7.376391227913056 PSNR Green: 7.82249135205217 PSNR Blue: 8.6876804304
42752
MD Red: 246 MD Green: 240 MD Blue: 238
IQI Red: 0.9999975461528052 Green: 0.999995545404802 Blue: 0.9999815504908023
CF between original and encrypted Red: -2.0847909257067586e-05 Green: 0.00181016
50656634962 Blue: -0.00032006429378566783
CF btween original and decrypted Red: 0.9999860054700207 Green: 0.99998514548946
58 Blue: 0.9999887555141813
```

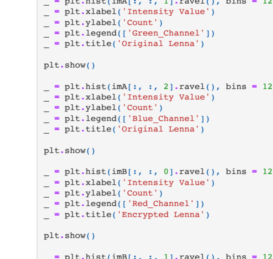

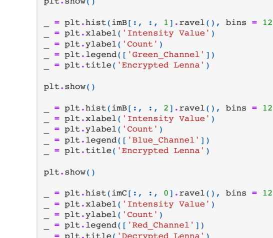

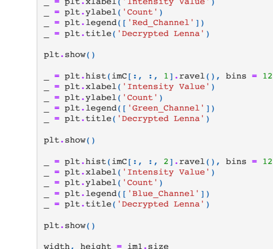

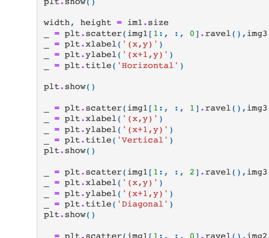

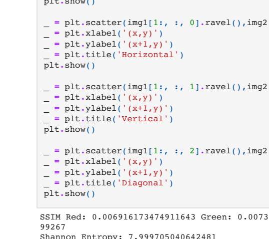

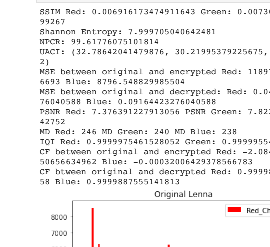

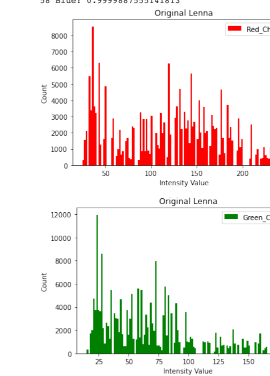

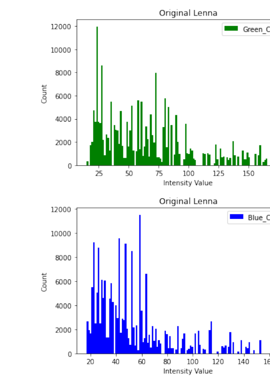

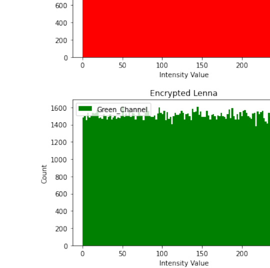

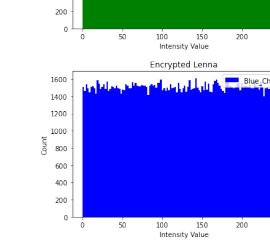

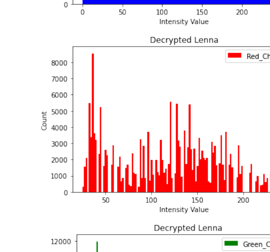

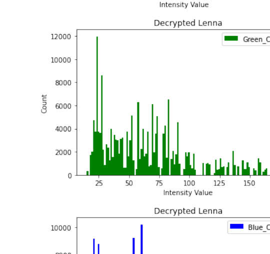

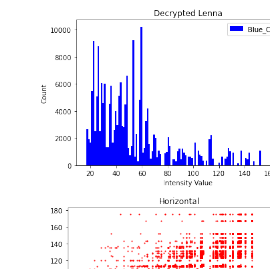

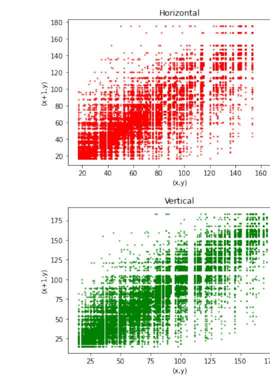

In [3]:

```python
imA = io.imread("~/Desktop/Paper_Files/Images_Analysis/img/Baboon.png")
imB = io.imread("~/Desktop/Paper_Files/Images_Analysis/img/Baboon_E.png")

im1 = Image.open("/Users/banan/Desktop/Paper_Files/Images_Analysis/img/Baboon.pn
im2 = Image.open("/Users/banan/Desktop/Paper_Files/Images_Analysis/img/Baboon_E.
im3 = Image.open("/Users/banan/Desktop/Paper_Files/Images_Analysis/img/Baboon_D.


img1 = cv2.imread("/Users/banan/Desktop/Paper_Files/Images_Analysis/img/Baboon.p
img2 = cv2.imread("/Users/banan/Desktop/Paper_Files/Images_Analysis/img/Baboon_E
img3 = cv2.imread("/Users/banan/Desktop/Paper_Files/Images_Analysis/img/Baboon_D




(scorer,scoreg,scoreb) = metrics.structural_similarity(img1[:,:,0], img2[:,:,0],


entropy  = measure.shannon_entropy(img2)
npcr  = npcrv(im1, im2)
uaci = uaciv(im1, im2)
mser,mseg,mseb  = msecalc(img1[:,:,0], img2[:,:,0]),msecalc(img1[:,:,1], img2[:,
psnrr,psnrg,psnrb = cv2.PSNR(img1[:,:,0], img2[:,:,0]),cv2.PSNR(img1[:,:,1], img

msedr,msedg,msedb  = msecalc(img1[:,:,0], img3[:,:,0]),msecalc(img1[:,:,1], img3
iqir,iqig,iqib = uqi(img1[:,:,0],img3[:,:,0]),uqi(img1[:,:,1],img3[:,:,1]), uqi(
mdr, mdg, mdb = mdv(im1,im2)
cfr, cfg, cfb = np.corrcoef(img1[:,:,0].flat, img3[:,:,0].flat)[0][1], np.corrco
cfer, cfeg, cfeb = np.corrcoef(img1[:,:,0].flat, img2[:,:,0].flat)[0][1], np.cor


print("SSIM Red: {}".format(scorer[0])+" Green: {}".format(scoreg[0])+" Blue: {}
print("Shannon Entropy: {}".format(entropy))
print("NPCR: {}".format(npcr))
print("UACI: {}".format(uaci))
print("MSE between original and encrypted Red: {}".format(mser)+" Green: {}".for
print("MSE between original and decrypted: Red: {}".format(msedr)+" Green: {}".f
print("PSNR Red: {}".format(psnrr)+" PSNR Green: {}".format(psnrg)+" PSNR Blue:
print("MD Red: {}".format(mdr)+" MD Green: {}".format(mdg)+" MD Blue: {}".format
print("IQI Red: {}".format(iqir)+" Green: {}".format(iqig)+" Blue: {}".format(iq
print("CF between original and encrypted Red: {}".format(cfer)+" Green: {}".form

_ = plt.hist(imA[:, :, 0].ravel(), bins = 128, color = 'red', alpha = 1)
_ = plt.xlabel('Intensity Value')
_ = plt.ylabel('Count')
_ = plt.legend(['Red_Channel'])
_ = plt.title('Original Baboon')

plt.show()

_ = plt.hist(imA[:, :, 1].ravel(), bins = 128, color = 'Green', alpha = 1)
_ = plt.xlabel('Intensity Value')
_ = plt.ylabel('Count')
_ = plt.legend(['Green_Channel'])
_ = plt.title('Original Baboon')

plt.show()

_ = plt.hist(imA[:, :, 2].ravel(), bins = 128, color = 'blue', alpha = 1)
_ = plt.xlabel('Intensity Value')
_ = plt.ylabel('Count')
_ = plt.legend(['Blue_Channel'])
_ = plt.title('Original Baboon')

plt.show()

_ = plt.hist(imB[:, :, 0].ravel(), bins = 128, color = 'red', alpha = 1)
_ = plt.xlabel('Intensity Value')
_ = plt.ylabel('Count')
_ = plt.legend(['Red_Channel'])
_ = plt.title('Encrypted Baboon')

plt.show()

_ = plt.hist(imB[:, :, 1].ravel(), bins = 128, color = 'Green', alpha = 1)
_ = plt.xlabel('Intensity Value')
_ = plt.ylabel('Count')
_ = plt.legend(['Green_Channel'])
_ = plt.title('Encrypted Baboon')

plt.show()

_ = plt.hist(imB[:, :, 2].ravel(), bins = 128, color = 'blue', alpha = 1)
_ = plt.xlabel('Intensity Value')
_ = plt.ylabel('Count')
_ = plt.legend(['Blue_Channel'])
_ = plt.title('Encrypted Baboon')

plt.show()

imC = io.imread("~/Desktop/Paper_Files/Images_Analysis/img/Baboon_D.png")

_ = plt.hist(imC[:, :, 0].ravel(), bins = 128, color = 'red', alpha = 1)
_ = plt.xlabel('Intensity Value')
_ = plt.ylabel('Count')
_ = plt.legend(['Red_Channel'])
_ = plt.title('Decrypted Baboon')

plt.show()

[illegible]
```

[illegible]

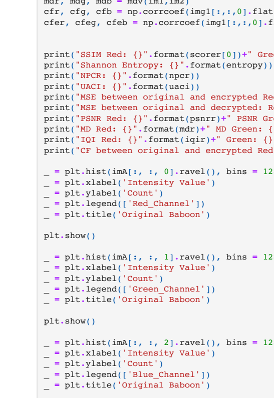

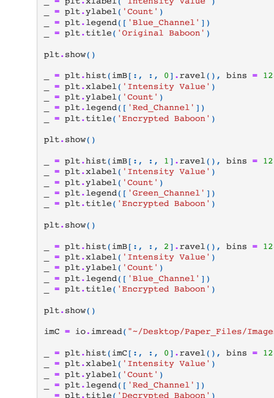

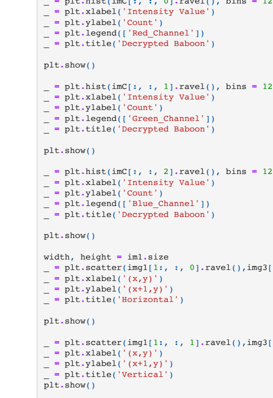

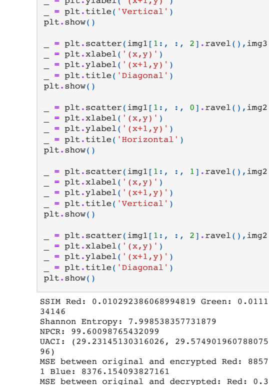

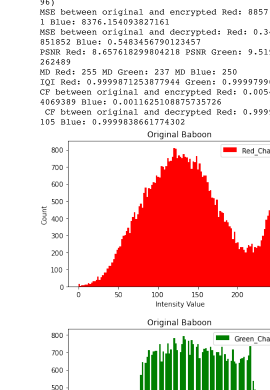

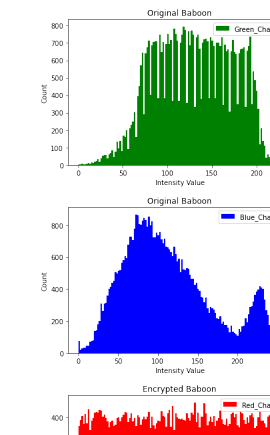

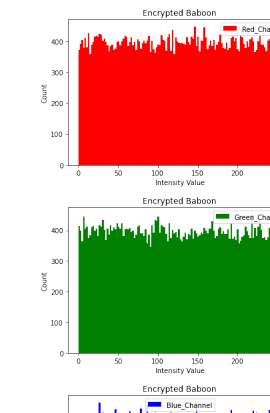

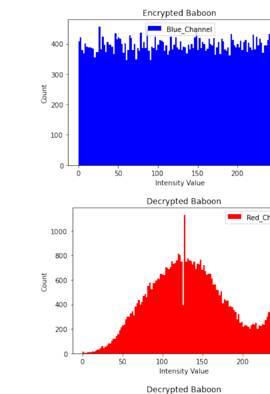

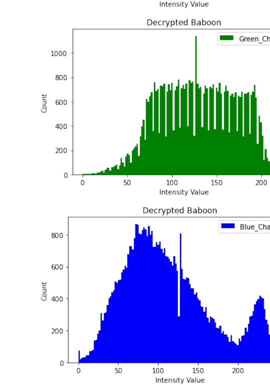

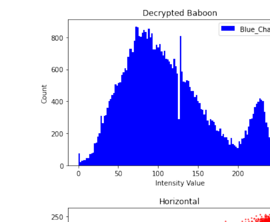

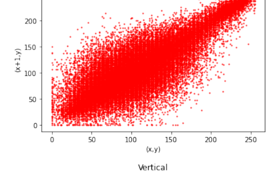

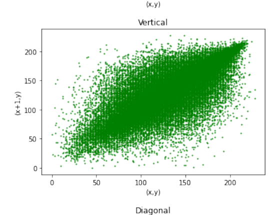

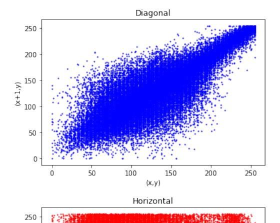

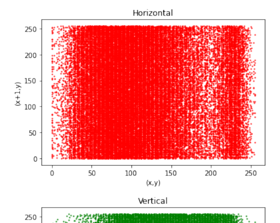

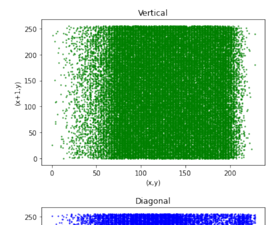

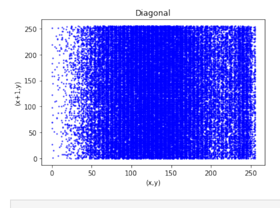

In [4]:

```
imA = io.imread("~/Desktop/Paper_Files/Images_Analysis/img/Cat1.png")
imB = io.imread("~/Desktop/Paper_Files/Images_Analysis/img/Cat1_E.png")

im1 = Image.open("/Users/banan/Desktop/Paper_Files/Images_Analysis/img/Cat1.png"
im2 = Image.open("/Users/banan/Desktop/Paper_Files/Images_Analysis/img/Cat1_E.pn

img1 = cv2.imread("/Users/banan/Desktop/Paper_Files/Images_Analysis/img/Cat1.png
img2 = cv2.imread("/Users/banan/Desktop/Paper_Files/Images_Analysis/img/Cat1_E.p
img3 = cv2.imread("/Users/banan/Desktop/Paper_Files/Images_Analysis/img/Cat1_D.p



(scorer,scoreg,scoreb) = metrics.structural_similarity(img1[:,:,0], img2[:,:,0],


entropy  = measure.shannon_entropy(img2)
npcr  = npcrv(im1, im2)
uaci = uaciv(im1, im2)
mser,mseg,mseb  = msecalc(img1[:,:,0], img2[:,:,0]),msecalc(img1[:,:,1], img2[:,
psnrr,psnrg,psnrb = cv2.PSNR(img1[:,:,0], img2[:,:,0]),cv2.PSNR(img1[:,:,1], img
msedr,msedg,msedb  = msecalc(img1[:,:,0], img3[:,:,0]),msecalc(img1[:,:,1], img3
iqir,iqig,iqib = uqi(img1[:,:,0],img3[:,:,0]),uqi(img1[:,:,1],img3[:,:,1]), uqi(
mdr, mdg, mdb = mdv(im1,im2)
cfr, cfg, cfb = np.corrcoef(img1[:,:,0].flat, img3[:,:,0].flat)[0][1], np.corrco
cfer, cfeg, cfeb = np.corrcoef(img1[:,:,0].flat, img2[:,:,0].flat)[0][1], np.cor


print("SSIM Red: {}".format(scorer[0])+" Green: {}".format(scoreg[0])+" Blue: {}
print("Shannon Entropy: {}".format(entropy))
print("NPCR: {}".format(npcr))
print("UACI: {}".format(uaci))
print("MSE between original and encrypted Red: {}".format(mser)+" Green: {}".for
print("MSE between original and decrypted: Red: {}".format(msedr)+" Green: {}".f
print("PSNR Red: {}".format(psnrr)+" PSNR Green: {}".format(psnrg)+" PSNR Blue:
print("MD Red: {}".format(mdr)+" MD Green: {}".format(mdg)+" MD Blue: {}".format
print("IQI Red: {}".format(iqir)+" Green: {}".format(iqig)+" Blue: {}".format(iq
print("CF between original and encrypted Red: {}".format(cfer)+" Green: {}".form
_ = plt.hist(imA[:, :, 0].ravel(), bins = 128, color = 'red', alpha = 1)
_ = plt.xlabel('Intensity Value')
_ = plt.ylabel('Count')
_ = plt.legend(['Red_Channel'])
_ = plt.title('Original Cat1')

plt.show()

_ = plt.hist(imA[:, :, 1].ravel(), bins = 128, color = 'Green', alpha = 1)
_ = plt.xlabel('Intensity Value')
_ = plt.ylabel('Count')
_ = plt.legend(['Green_Channel'])
_ = plt.title('Original Cat1')

plt.show()

_ = plt.hist(imA[:, :, 2].ravel(), bins = 128, color = 'blue', alpha = 1)
_ = plt.xlabel('Intensity Value')
_ = plt.ylabel('Count')
_ = plt.legend(['Blue_Channel'])
_ = plt.title('Original Cat1')

plt.show()

_ = plt.hist(imB[:, :, 0].ravel(), bins = 128, color = 'red', alpha = 1)
_ = plt.xlabel('Intensity Value')
_ = plt.ylabel('Count')
_ = plt.legend(['Red_Channel'])
_ = plt.title('Encrypted Cat1')

plt.show()

_ = plt.hist(imB[:, :, 1].ravel(), bins = 128, color = 'Green', alpha = 1)
_ = plt.xlabel('Intensity Value')
_ = plt.ylabel('Count')
_ = plt.legend(['Green_Channel'])
_ = plt.title('Encrypted Cat1')

plt.show()

_ = plt.hist(imB[:, :, 2].ravel(), bins = 128, color = 'blue', alpha = 1)
_ = plt.xlabel('Intensity Value')
_ = plt.ylabel('Count')
_ = plt.legend(['Blue_Channel'])
_ = plt.title('Encrypted Cat1')

plt.show()

imC = io.imread("~/Desktop/Paper_Files/Images_Analysis/img/Cat1_D.png")

_ = plt.hist(imC[:, :, 0].ravel(), bins = 128, color = 'red', alpha = 1)
_ = plt.xlabel('Intensity Value')
_ = plt.ylabel('Count')
_ = plt.legend(['Red_Channel'])
_ = plt.title('Decrypted Cat1')

plt.show()

_ = plt.hist(imC[:, :, 1].ravel(), bins = 128, color = 'Green', alpha = 1)
_ = plt.xlabel('Intensity Value')
_ = plt.ylabel('Count')
_ = plt.legend(['Green_Channel'])
_ = plt.title('Decrypted Cat1')

plt.show()

_ = plt.hist(imC[:, :, 2].ravel(), bins = 128, color = 'blue', alpha = 1)
_ = plt.xlabel('Intensity Value')
_ = plt.ylabel('Count')
_ = plt.legend(['Blue_Channel'])
_ = plt.title('Decrypted Cat1')

plt.show()

width, height = im1.size
_ = plt.scatter(img1[1:, :, 0].ravel(),img3[:height-1, :, 0].ravel(), s=1, c = '
_ = plt.xlabel('(x,y)')
_ = plt.ylabel('(x+1,y)')
_ = plt.title('Horizontal')

plt.show()

_ = plt.scatter(img1[1:, :, 1].ravel(),img3[:height-1, :, 1].ravel(), s=1, c = '
_ = plt.xlabel('(x,y)')
_ = plt.ylabel('(x+1,y)')
_ = plt.title('Vertical')
plt.show()

_ = plt.scatter(img1[1:, :, 2].ravel(),img3[:height-1, :, 2].ravel(), s=1, c = '
_ = plt.xlabel('(x,y)')
_ = plt.ylabel('(x+1,y)')
_ = plt.title('Diagonal')
plt.show()

_ = plt.scatter(img1[1:, :, 0].ravel(),img2[:height-1, :, 0].ravel(), s=1, c = '
_ = plt.xlabel('(x,y)')
_ = plt.ylabel('(x+1,y)')
_ = plt.title('Horizontal')
plt.show()

_ = plt.scatter(img1[1:, :, 1].ravel(),img2[:height-1, :, 1].ravel(), s=1, c = '
_ = plt.xlabel('(x,y)')
_ = plt.ylabel('(x+1,y)')
_ = plt.title('Vertical')
plt.show()

_ = plt.scatter(img1[1:, :, 2].ravel(),img2[:height-1, :, 2].ravel(), s=1, c = '
_ = plt.xlabel('(x,y)')
_ = plt.ylabel('(x+1,y)')
_ = plt.title('Diagonal')
plt.show()
```

```
SSIM Red: 0.002305237688667445 Green: 0.009728682743743616 Blue: 0.0106497108403
76749
Shannon Entropy: 7.9984006898655124
NPCR: 99.60583333333334
UACI: (31.922924836604054, 31.512460784315277, 31.69100980392455, 31.69100980392
455)
MSE between original and encrypted Red: 10345.664325 Green: 9790.390975 Blue: 96
88.401375
MSE between original and decrypted: Red: 0.29475 Green: 0.344975 Blue: 0.3361
PSNR Red: 7.983219776671842 PSNR Green: 8.222803253574583 PSNR Blue: 8.268282382
33242
MD Red: 255 MD Green: 255 MD Blue: 255
IQI Red: 0.9999895156061888 Green: 0.9999880744953329 Blue: 0.999988655869636
CF between original and encrypted Red: -0.003416515593387978 Green: 0.0079298255
98479505 Blue: 0.009330855007717468
 CF btween original and decrypted Red: 0.9999918146470731 Green: 0.9999919617588
44 Blue: 0.9999911161840201
```

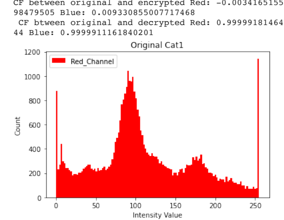

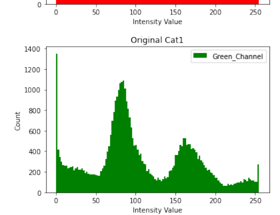

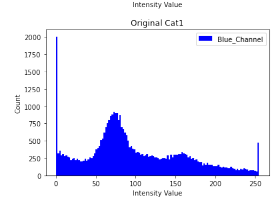

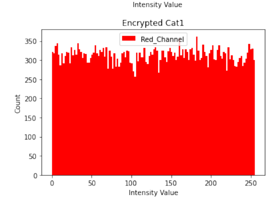

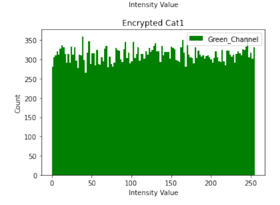

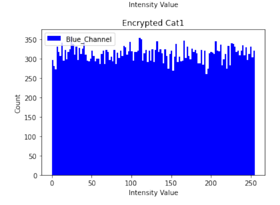

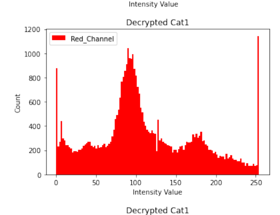

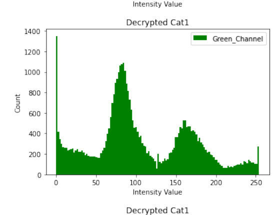

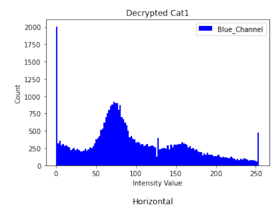

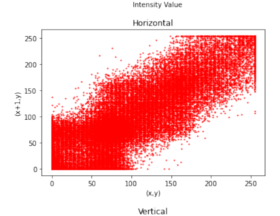

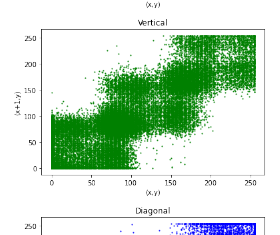

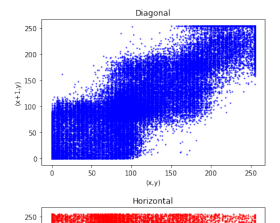

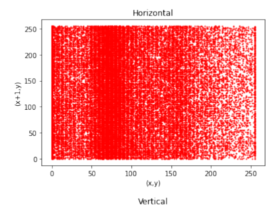

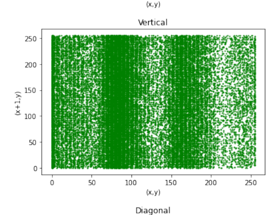

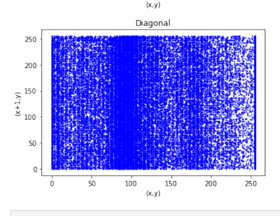

In [5]:

```
imA = io.imread("~/Desktop/Paper_Files/Images_Analysis/img/Cat2.png")
imB = io.imread("~/Desktop/Paper_Files/Images_Analysis/img/Cat2_E.png")

im1 = Image.open("/Users/banan/Desktop/Paper_Files/Images_Analysis/img/Cat2.png"
im2 = Image.open("/Users/banan/Desktop/Paper_Files/Images_Analysis/img/Cat2_E.pn

img1 = cv2.imread("/Users/banan/Desktop/Paper_Files/Images_Analysis/img/Cat2.png
img2 = cv2.imread("/Users/banan/Desktop/Paper_Files/Images_Analysis/img/Cat2_E.p
img3 = cv2.imread("/Users/banan/Desktop/Paper_Files/Images_Analysis/img/Cat2_D.p



(scorer,scoreg,scoreb) = metrics.structural_similarity(img1[:,:,0], img2[:,:,0],

entropy  = measure.shannon_entropy(img2)
npcr  = npcrv(im1, im2)
uaci = uaciv(im1, im2)
mser,mseg,mseb  = msecalc(img1[:,:,0], img2[:,:,0]),msecalc(img1[:,:,1], img2[:,
psnrr,psnrg,psnrb = cv2.PSNR(img1[:,:,0], img2[:,:,0]),cv2.PSNR(img1[:,:,1], img

msedr,msedg,msedb  = msecalc(img1[:,:,0], img3[:,:,0]),msecalc(img1[:,:,1], img3
iqir,iqig,iqib = uqi(img1[:,:,0],img3[:,:,0]),uqi(img1[:,:,1],img3[:,:,1]), uqi(
mdr, mdg, mdb = mdv(im1,im2)
cfr, cfg, cfb = np.corrcoef(img1[:,:,0].flat, img3[:,:,0].flat)[0][1], np.corrco
cfer, cfeg, cfeb = np.corrcoef(img1[:,:,0].flat, img2[:,:,0].flat)[0][1], np.cor


print("SSIM Red: {}".format(scorer[0])+" Green: {}".format(scoreg[0])+" Blue: {}
print("Shannon Entropy: {}".format(entropy))
print("NPCR: {}".format(npcr))
print("UACI: {}".format(uaci))
print("MSE between original and encrypted Red: {}".format(mser)+" Green: {}".for
print("MSE between original and decrypted: Red: {}".format(msedr)+" Green: {}".f
print("PSNR Red: {}".format(psnrr)+" PSNR Green: {}".format(psnrg)+" PSNR Blue:
print("MD Red: {}".format(mdr)+" MD Green: {}".format(mdg)+" MD Blue: {}".format
print("IQI Red: {}".format(iqir)+" Green: {}".format(iqig)+" Blue: {}".format(iq
print("CF between original and encrypted Red: {}".format(cfer)+" Green: {}".form

_ = plt.hist(imA[:, :, 0].ravel(), bins = 128, color = 'red', alpha = 1)
_ = plt.xlabel('Intensity Value')
_ = plt.ylabel('Count')
_ = plt.legend(['Red_Channel'])
_ = plt.title('Original Cat2')

plt.show()

_ = plt.hist(imA[:, :, 1].ravel(), bins = 128, color = 'Green', alpha = 1)
_ = plt.xlabel('Intensity Value')
_ = plt.ylabel('Count')
_ = plt.legend(['Green_Channel'])
_ = plt.title('Original Cat2')

plt.show()

_ = plt.hist(imA[:, :, 2].ravel(), bins = 128, color = 'blue', alpha = 1)
_ = plt.xlabel('Intensity Value')
_ = plt.ylabel('Count')
_ = plt.legend(['Blue_Channel'])
_ = plt.title('Original Cat2')

plt.show()

_ = plt.hist(imB[:, :, 0].ravel(), bins = 128, color = 'red', alpha = 1)
_ = plt.xlabel('Intensity Value')
_ = plt.ylabel('Count')
_ = plt.legend(['Red_Channel'])
_ = plt.title('Encrypted Cat2')

plt.show()

_ = plt.hist(imB[:, :, 1].ravel(), bins = 128, color = 'Green', alpha = 1)
_ = plt.xlabel('Intensity Value')
_ = plt.ylabel('Count')
_ = plt.legend(['Green_Channel'])
_ = plt.title('Encrypted Cat2')

plt.show()

_ = plt.hist(imB[:, :, 2].ravel(), bins = 128, color = 'blue', alpha = 1)
_ = plt.xlabel('Intensity Value')
_ = plt.ylabel('Count')
_ = plt.legend(['Blue_Channel'])
_ = plt.title('Encrypted Cat2')

plt.show()

imC = io.imread("~/Desktop/Paper_Files/Images_Analysis/img/Cat2_D.png")

_ = plt.hist(imC[:, :, 0].ravel(), bins = 128, color = 'red', alpha = 1)
_ = plt.xlabel('Intensity Value')
_ = plt.ylabel('Count')
_ = plt.legend(['Red_Channel'])
_ = plt.title('Decrypted Cat2')

plt.show()

_ = plt.hist(imC[:, :, 1].ravel(), bins = 128, color = 'Green', alpha = 1)
_ = plt.xlabel('Intensity Value')
_ = plt.ylabel('Count')
_ = plt.legend(['Green_Channel'])
_ = plt.title('Decrypted Cat2')

plt.show()

_ = plt.hist(imC[:, :, 2].ravel(), bins = 128, color = 'blue', alpha = 1)
_ = plt.xlabel('Intensity Value')
_ = plt.ylabel('Count')
_ = plt.legend(['Blue_Channel'])
_ = plt.title('Decrypted Cat2')

plt.show()

width, height = im1.size
_ = plt.scatter(img1[1:, :, 0].ravel(),img3[:height-1, :, 0].ravel(), s=1, c = '
_ = plt.xlabel('(x,y)')
_ = plt.ylabel('(x+1,y)')
_ = plt.title('Horizontal')

plt.show()

_ = plt.scatter(img1[1:, :, 1].ravel(),img3[:height-1, :, 1].ravel(), s=1, c = '
_ = plt.xlabel('(x,y)')
_ = plt.ylabel('(x+1,y)')
_ = plt.title('Vertical')
plt.show()

_ = plt.scatter(img1[1:, :, 2].ravel(),img3[:height-1, :, 2].ravel(), s=1, c = '
_ = plt.xlabel('(x,y)')
_ = plt.ylabel('(x+1,y)')
_ = plt.title('Diagonal')
plt.show()

_ = plt.scatter(img1[1:, :, 0].ravel(),img2[:height-1, :, 0].ravel(), s=1, c = '
_ = plt.xlabel('(x,y)')
_ = plt.ylabel('(x+1,y)')
_ = plt.title('Horizontal')
plt.show()

_ = plt.scatter(img1[1:, :, 1].ravel(),img2[:height-1, :, 1].ravel(), s=1, c = '
_ = plt.xlabel('(x,y)')
_ = plt.ylabel('(x+1,y)')
_ = plt.title('Vertical')
plt.show()

_ = plt.scatter(img1[1:, :, 2].ravel(),img2[:height-1, :, 2].ravel(), s=1, c = '
_ = plt.xlabel('(x,y)')
_ = plt.ylabel('(x+1,y)')
_ = plt.title('Diagonal')
plt.show()
```

```
SSIM Red: 0.007443149185711921 Green: 0.009251846648637608 Blue: 0.0089140630018
64703
Shannon Entropy: 7.993050668755496
NPCR: 99.62683062209128
UACI: (34.79370198188592, 36.060699673750854, 34.257370836131756, 34.25737083613
1756)
MSE between original and encrypted Red: 11322.603279108493 Green: 11445.33979761
7522 Blue: 12637.4770078135
MSE between original and decrypted: Red: 0.5454335852440118 Green: 0.68336108620
4688 Blue: 0.8257461252721916
PSNR Red: 7.5913407007839595 PSNR Green: 7.544516700137823 PSNR Blue: 7.11419982
4084221
MD Red: 255 MD Green: 255 MD Blue: 255
IQI Red: 0.9999835451988389 Green: 0.9999800112220162 Blue: 0.9999781161946532
CF between original and encrypted Red: -0.003818613358834323 Green: -0.006017340
014071938 Blue: -0.0045732295625578595
 CF btween original and decrypted Red: 0.9999927218731836 Green: 0.9999908895820
382 Blue: 0.9999906310916014
```

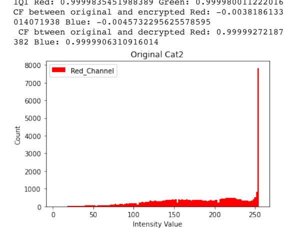

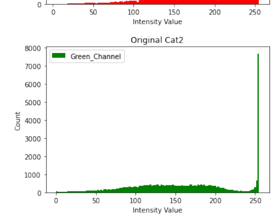

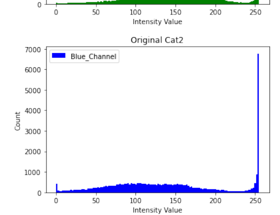

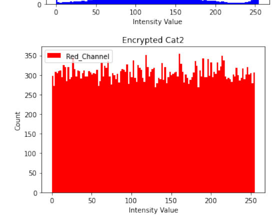

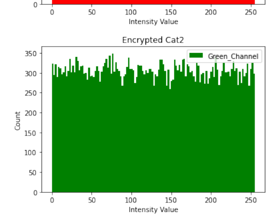

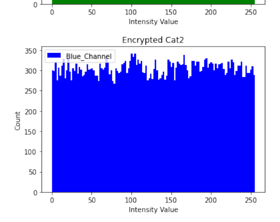

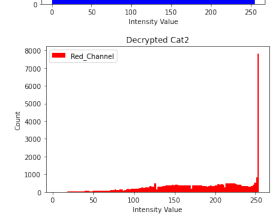

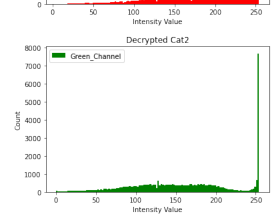

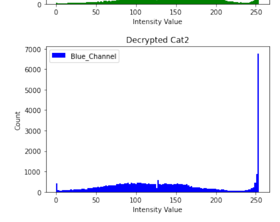

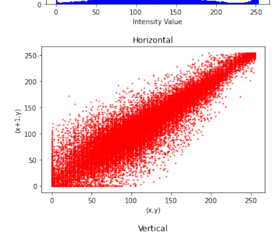

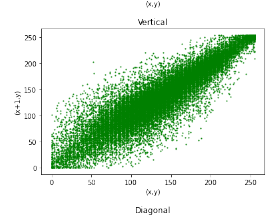

Diagonal

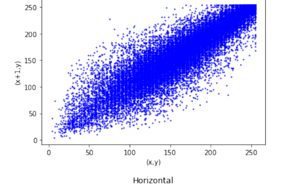

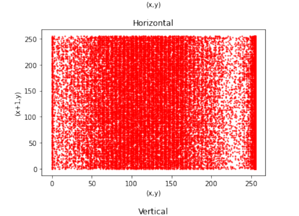

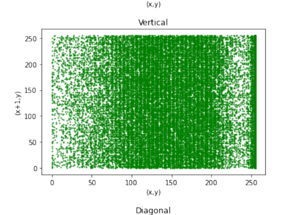

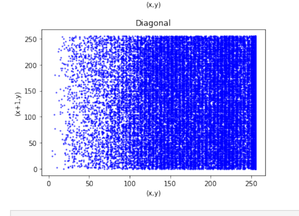

In [6]:

```
imA = io.imread("~/Desktop/Paper_Files/Images_Analysis/img/Dog.png")
imB = io.imread("~/Desktop/Paper_Files/Images_Analysis/img/Dog_E.png")

im1 = Image.open("/Users/banan/Desktop/Paper_Files/Images_Analysis/img/Dog.png")
im2 = Image.open("/Users/banan/Desktop/Paper_Files/Images_Analysis/img/Dog_E.png

img1 = cv2.imread("/Users/banan/Desktop/Paper_Files/Images_Analysis/img/Dog.png"
img2 = cv2.imread("/Users/banan/Desktop/Paper_Files/Images_Analysis/img/Dog_E.pn
img3 = cv2.imread("/Users/banan/Desktop/Paper_Files/Images_Analysis/img/Dog_D.pn




(scorer,scoreg,scoreb) = metrics.structural_similarity(img1[:,:,0], img2[:,:,0],

entropy  = measure.shannon_entropy(img2)
npcr  = npcrv(im1, im2)
uaci = uaciv(im1, im2)
mser,mseg,mseb  = msecalc(img1[:,:,0], img2[:,:,0]),msecalc(img1[:,:,1], img2[:,
psnrr,psnrg,psnrb = cv2.PSNR(img1[:,:,0], img2[:,:,0]),cv2.PSNR(img1[:,:,1], img

msedr,msedg,msedb  = msecalc(img1[:,:,0], img3[:,:,0]),msecalc(img1[:,:,1], img3
iqir,iqig,iqib = uqi(img1[:,:,0],img3[:,:,0]),uqi(img1[:,:,1],img3[:,:,1]), uqi(
mdr, mdg, mdb = mdv(im1,im2)
cfr, cfg, cfb = np.corrcoef(img1[:,:,0].flat, img3[:,:,0].flat)[0][1], np.corrco
cfer, cfeg, cfeb = np.corrcoef(img1[:,:,0].flat, img2[:,:,0].flat)[0][1], np.cor


print("SSIM Red: {}".format(scorer[0])+" Green: {}".format(scoreg[0])+" Blue: {}
print("Shannon Entropy: {}".format(entropy))
print("NPCR: {}".format(npcr))
print("UACI: {}".format(uaci))
print("MSE between original and encrypted Red: {}".format(mser)+" Green: {}".for
print("MSE between original and decrypted: Red: {}".format(msedr)+" Green: {}".f
print("PSNR Red: {}".format(psnrr)+" PSNR Green: {}".format(psnrg)+" PSNR Blue: 
print("MD Red: {}".format(mdr)+" MD Green: {}".format(mdg)+" MD Blue: {}".format
print("IQI Red: {}".format(iqir)+" Green: {}".format(iqig)+" Blue: {}".format(iq
print("CF between original and encrypted Red: {}".format(cfer)+" Green: {}".form

_ = plt.hist(imA[:, :, 0].ravel(), bins = 128, color = 'red', alpha = 1)
_ = plt.xlabel('Intensity Value')
_ = plt.ylabel('Count')
_ = plt.legend(['Red_Channel'])
_ = plt.title('Original Dog')

plt.show()

_ = plt.hist(imA[:, :, 1].ravel(), bins = 128, color = 'Green', alpha = 1)
_ = plt.xlabel('Intensity Value')
_ = plt.ylabel('Count')
_ = plt.legend(['Green_Channel'])
_ = plt.title('Original Dog')

plt.show()

_ = plt.hist(imA[:, :, 2].ravel(), bins = 128, color = 'blue', alpha = 1)
_ = plt.xlabel('Intensity Value')
_ = plt.ylabel('Count')
_ = plt.legend(['Blue_Channel'])
_ = plt.title('Original Dog')

plt.show()

_ = plt.hist(imB[:, :, 0].ravel(), bins = 128, color = 'red', alpha = 1)
_ = plt.xlabel('Intensity Value')
_ = plt.ylabel('Count')
_ = plt.legend(['Red_Channel'])
_ = plt.title('Encrypted Dog')

plt.show()

_ = plt.hist(imB[:, :, 1].ravel(), bins = 128, color = 'Green', alpha = 1)
_ = plt.xlabel('Intensity Value')
_ = plt.ylabel('Count')
_ = plt.legend(['Green_Channel'])
_ = plt.title('Encrypted Dog')

plt.show()

_ = plt.hist(imB[:, :, 2].ravel(), bins = 128, color = 'blue', alpha = 1)
_ = plt.xlabel('Intensity Value')
_ = plt.ylabel('Count')
_ = plt.legend(['Blue_Channel'])
_ = plt.title('Encrypted Dog')

plt.show()

imC = io.imread("~/Desktop/Paper_Files/Images_Analysis/img/Dog_D.png")

_ = plt.hist(imC[:, :, 0].ravel(), bins = 128, color = 'red', alpha = 1)
_ = plt.xlabel('Intensity Value')
_ = plt.ylabel('Count')
_ = plt.legend(['Red_Channel'])
_ = plt.title('Decrypted Dog')

plt.show()

_ = plt.hist(imC[:, :, 1].ravel(), bins = 128, color = 'Green', alpha = 1)
_ = plt.xlabel('Intensity Value')
_ = plt.ylabel('Count')
_ = plt.legend(['Green_Channel'])
_ = plt.title('Decrypted Dog')

plt.show()

_ = plt.hist(imC[:, :, 2].ravel(), bins = 128, color = 'blue', alpha = 1)
_ = plt.xlabel('Intensity Value')
_ = plt.ylabel('Count')
_ = plt.legend(['Blue_Channel'])
_ = plt.title('Decrypted Dog')

plt.show()

width, height = im1.size
_ = plt.scatter(img1[1:, :, 0].ravel(),img3[:height-1, :, 0].ravel(), s=1, c = '
_ = plt.xlabel('(x,y)')
_ = plt.ylabel('(x+1,y)')
_ = plt.title('Horizontal')

plt.show()

_ = plt.scatter(img1[1:, :, 1].ravel(),img3[:height-1, :, 1].ravel(), s=1, c = '
_ = plt.xlabel('(x,y)')
_ = plt.ylabel('(x+1,y)')
_ = plt.title('Vertical')
plt.show()

_ = plt.scatter(img1[1:, :, 2].ravel(),img3[:height-1, :, 2].ravel(), s=1, c = '
_ = plt.xlabel('(x,y)')
_ = plt.ylabel('(x+1,y)')
_ = plt.title('Diagonal')
plt.show()

_ = plt.scatter(img1[1:, :, 0].ravel(),img2[:height-1, :, 0].ravel(), s=1, c = '
_ = plt.xlabel('(x,y)')
_ = plt.ylabel('(x+1,y)')
_ = plt.title('Horizontal')
plt.show()

_ = plt.scatter(img1[1:, :, 1].ravel(),img2[:height-1, :, 1].ravel(), s=1, c = '
_ = plt.xlabel('(x,y)')
_ = plt.ylabel('(x+1,y)')
_ = plt.title('Vertical')
plt.show()

_ = plt.scatter(img1[1:, :, 2].ravel(),img2[:height-1, :, 2].ravel(), s=1, c = '
_ = plt.xlabel('(x,y)')
_ = plt.ylabel('(x+1,y)')
_ = plt.title('Diagonal')
plt.show()
```

```
SSIM Red: 0.008667340136903203 Green: 0.009156726986227555 Blue: 0.0103060603862
25823
Shannon Entropy: 7.998787588255452
NPCR: 99.619708994709
UACI: (29.9900352733747, 29.73211173358832, 29.895845004674914, 29.8958450046749
14)
MSE between original and encrypted Red: 8922.190416666666 Green: 8565.8227777777
77 Blue: 8499.715257936508
MSE between original and decrypted: Red: 0.3203769841269841 Green: 0.39529761904
761906 Blue: 0.485694444444443
PSNR Red: 8.626088731856779 PSNR Green: 8.803112760314288 PSNR Blue: 8.836759838
56943
MD Red: 255 MD Green: 250 MD Blue: 250
IQI Red: 0.9999901167771715 Green: 0.9999875084016981 Blue: 0.9999830925542254
CF between original and encrypted Red: 0.002305843835957798 Green: -0.0033342914
90213059 Blue: 0.004846478033336193
 CF btween original and decrypted Red: 0.9999941671822141 Green: 0.9999895052963
794 Blue: 0.9999847975186994
```

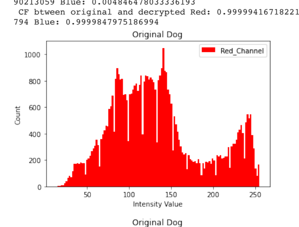

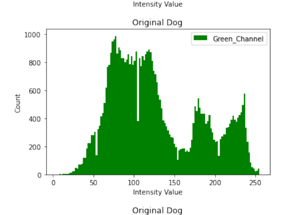

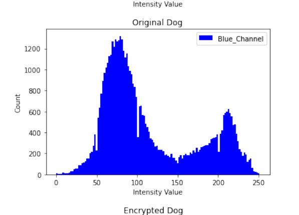

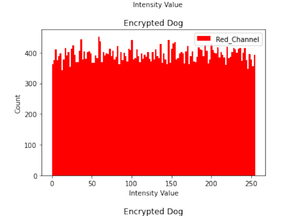

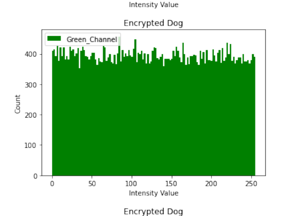

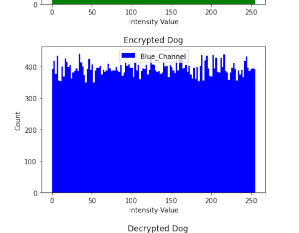

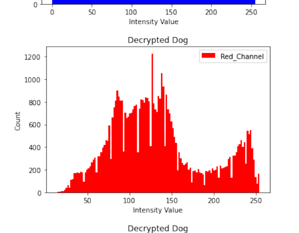

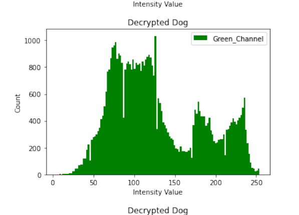

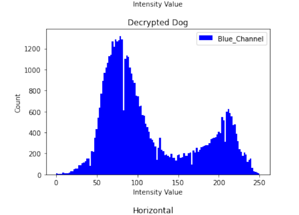

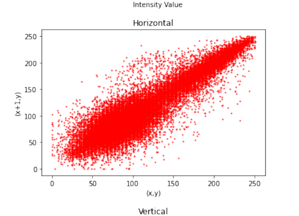

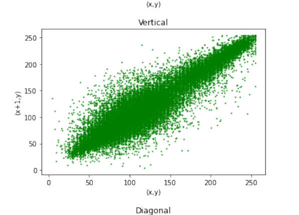

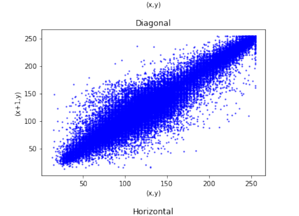

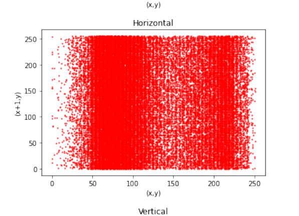

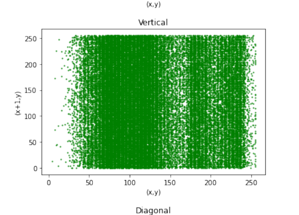

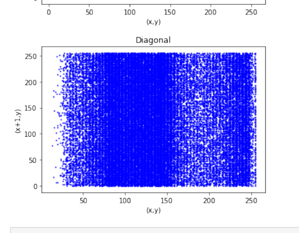

In [7]:

```
imA = io.imread("~/Desktop/Paper_Files/Images_Analysis/img/Eye.png")
imB = io.imread("~/Desktop/Paper_Files/Images_Analysis/img/Eye_E.png")

im1 = Image.open("/Users/banan/Desktop/Paper_Files/Images_Analysis/img/Eye.png")
im2 = Image.open("/Users/banan/Desktop/Paper_Files/Images_Analysis/img/Eye_E.png

img1 = cv2.imread("/Users/banan/Desktop/Paper_Files/Images_Analysis/img/Eye.png"
img2 = cv2.imread("/Users/banan/Desktop/Paper_Files/Images_Analysis/img/Eye_E.pn
img3 = cv2.imread("/Users/banan/Desktop/Paper_Files/Images_Analysis/img/Eye_D.pn




(scorer,scoreg,scoreb) = metrics.structural_similarity(img1[:,:,0], img2[:,:,0],

entropy  = measure.shannon_entropy(img2)
npcr  = npcrv(im1, im2)
uaci = uaciv(im1, im2)
mser,mseg,mseb  = msecalc(img1[:,:,0], img2[:,:,0]),msecalc(img1[:,:,1], img2[:,
psnrr,psnrg,psnrb = cv2.PSNR(img1[:,:,0], img2[:,:,0]),cv2.PSNR(img1[:,:,1], img

msedr,msedg,msedb  = msecalc(img1[:,:,0], img3[:,:,0]),msecalc(img1[:,:,1], img3
iqir,iqig,iqib = uqi(img1[:,:,0],img3[:,:,0]),uqi(img1[:,:,1],img3[:,:,1]), uqi(
mdr, mdg, mdb = mdv(im1,im2)
cfr, cfg, cfb = np.corrcoef(img1[:,:,0].flat, img3[:,:,0].flat)[0][1], np.corrco
cfer, cfeg, cfeb = np.corrcoef(img1[:,:,0].flat, img2[:,:,0].flat)[0][1], np.cor


print("SSIM Red: {}".format(scorer[0])+" Green: {}".format(scoreg[0])+" Blue: {}
print("Shannon Entropy: {}".format(entropy))
print("NPCR: {}".format(npcr))
print("UACI: {}".format(uaci))
print("MSE between original and encrypted Red: {}".format(mser)+" Green: {}".for
print("MSE between original and decrypted: Red: {}".format(msedr)+" Green: {}".f
print("PSNR Red: {}".format(psnrr)+" PSNR Green: {}".format(psnrg)+" PSNR Blue: 
print("MD Red: {}".format(mdr)+" MD Green: {}".format(mdg)+" MD Blue: {}".format
print("IQI Red: {}".format(iqir)+" Green: {}".format(iqig)+" Blue: {}".format(iq
print("CF between original and encrypted Red: {}".format(cfer)+" Green: {}".form

_ = plt.hist(imA[:, :, 0].ravel(), bins = 128, color = 'red', alpha = 1)
_ = plt.xlabel('Intensity Value')
_ = plt.ylabel('Count')
_ = plt.legend(['Red_Channel'])
_ = plt.title('Encrypted Eye')

plt.show()

_ = plt.hist(imA[:, :, 1].ravel(), bins = 128, color = 'Green', alpha = 1)
_ = plt.xlabel('Intensity Value')
_ = plt.ylabel('Count')
_ = plt.legend(['Green_Channel'])
_ = plt.title('Encrypted Eye')

plt.show()

_ = plt.hist(imA[:, :, 2].ravel(), bins = 128, color = 'blue', alpha = 1)
_ = plt.xlabel('Intensity Value')
_ = plt.ylabel('Count')
_ = plt.legend(['Blue_Channel'])
_ = plt.title('Encrypted Eye')

plt.show()

_ = plt.hist(imB[:, :, 0].ravel(), bins = 128, color = 'red', alpha = 1)
_ = plt.xlabel('Intensity Value')
_ = plt.ylabel('Count')
_ = plt.legend(['Red_Channel'])
_ = plt.title('Encrypted Eye')

plt.show()

_ = plt.hist(imB[:, :, 1].ravel(), bins = 128, color = 'Green', alpha = 1)
_ = plt.xlabel('Intensity Value')
_ = plt.ylabel('Count')
_ = plt.legend(['Green_Channel'])
_ = plt.title('Encrypted Eye')

plt.show()

_ = plt.hist(imB[:, :, 2].ravel(), bins = 128, color = 'blue', alpha = 1)
_ = plt.xlabel('Intensity Value')
_ = plt.ylabel('Count')
_ = plt.legend(['Blue_Channel'])
_ = plt.title('Encrypted Eye')

plt.show()

imC = io.imread("~/Desktop/Paper_Files/Images_Analysis/img/Eye_D.png")

_ = plt.hist(imC[:, :, 0].ravel(), bins = 128, color = 'red', alpha = 1)
_ = plt.xlabel('Intensity Value')
_ = plt.ylabel('Count')
_ = plt.legend(['Red_Channel'])
_ = plt.title('Decrypted Eye')

plt.show()

_ = plt.hist(imC[:, :, 1].ravel(), bins = 128, color = 'Green', alpha = 1)
_ = plt.xlabel('Intensity Value')
_ = plt.ylabel('Count')
_ = plt.legend(['Green_Channel'])
_ = plt.title('Decrypted Eye')

plt.show()

_ = plt.hist(imC[:, :, 2].ravel(), bins = 128, color = 'blue', alpha = 1)
_ = plt.xlabel('Intensity Value')
_ = plt.ylabel('Count')
_ = plt.legend(['Blue_Channel'])
_ = plt.title('Decrypted Eye')

plt.show()

width, height = im1.size
_ = plt.scatter(img1[1:, :, 0].ravel(),img3[:height-1, :, 0].ravel(), s=1, c = '
_ = plt.xlabel('(x,y)')
_ = plt.ylabel('(x+1,y)')
_ = plt.title('Horizontal')

plt.show()

_ = plt.scatter(img1[1:, :, 1].ravel(),img3[:height-1, :, 1].ravel(), s=1, c = '
_ = plt.xlabel('(x,y)')
_ = plt.ylabel('(x+1,y)')
_ = plt.title('Vertical')
plt.show()

_ = plt.scatter(img1[1:, :, 2].ravel(),img3[:height-1, :, 2].ravel(), s=1, c = '
_ = plt.xlabel('(x,y)')
_ = plt.ylabel('(x+1,y)')
_ = plt.title('Diagonal')
plt.show()

_ = plt.scatter(img1[1:, :, 0].ravel(),img2[:height-1, :, 0].ravel(), s=1, c = '
_ = plt.xlabel('(x,y)')
_ = plt.ylabel('(x+1,y)')
_ = plt.title('Horizontal')
plt.show()

_ = plt.scatter(img1[1:, :, 1].ravel(),img2[:height-1, :, 1].ravel(), s=1, c = '
_ = plt.xlabel('(x,y)')
_ = plt.ylabel('(x+1,y)')
_ = plt.title('Vertical')
plt.show()

_ = plt.scatter(img1[1:, :, 2].ravel(),img2[:height-1, :, 2].ravel(), s=1, c = '
_ = plt.xlabel('(x,y)')
_ = plt.ylabel('(x+1,y)')
_ = plt.title('Diagonal')
plt.show()
```

```
SSIM Red: 0.005462494988859452 Green: 0.010930830792027208 Blue: 0.0089449916397
78431
Shannon Entropy: 7.998823531979197
NPCR: 99.58317639673571
UACI: (31.992902773166534, 29.784210331972332, 27.243425279719602, 27.2434252797
19602)
MSE between original and encrypted Red: 14541.222109227872 Green: 6859.287156308
851 Blue: 8540.935687382298
MSE between original and decrypted: Red: 0.0020527306967984935 Green: 0.54314500
94161958 Blue: 0.8705649717514125
PSNR Red: 6.504794527637497 PSNR Green: 9.76801376380919 PSNR Blue: 8.8157490919
44951
MD Red: 255 MD Green: 255 MD Blue: 255
IQI Red: 0.9999997908519338 Green: 0.9999765834323603 Blue: 0.999971126873982
CF between original and encrypted Red: 0.005481886963299706 Green: -0.0049114411
48746785 Blue: -0.0040428844378981406
 CF btween original and decrypted Red: 0.999997294577343 Green: 0.99995737507388
99 Blue: 0.9999851661315189
```

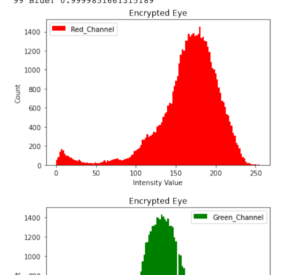

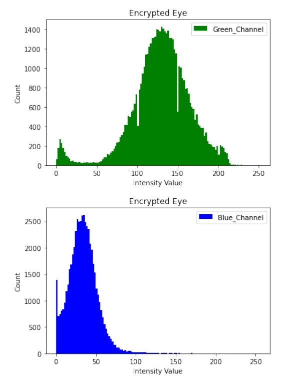

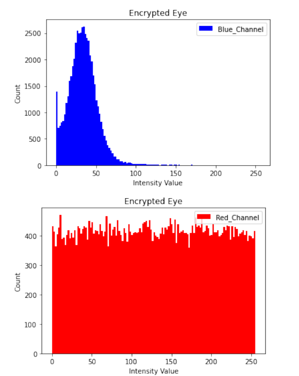

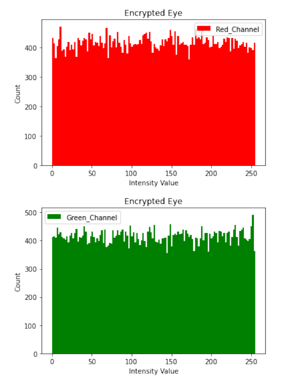

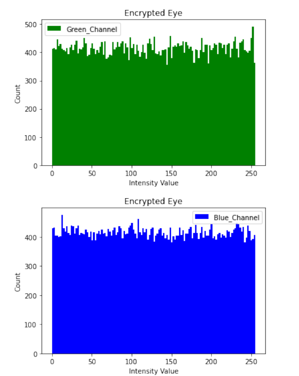

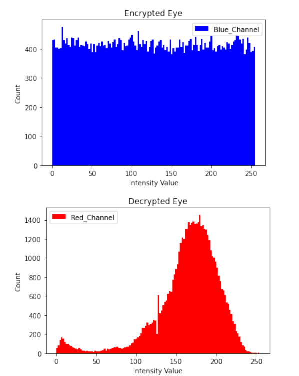

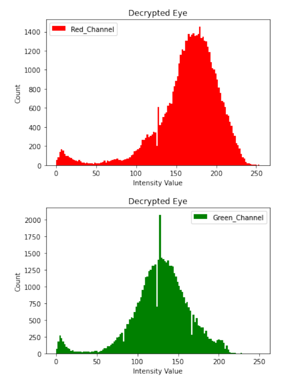

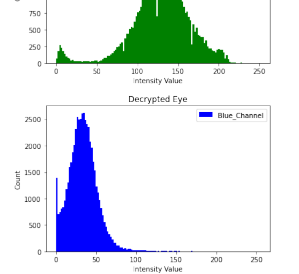

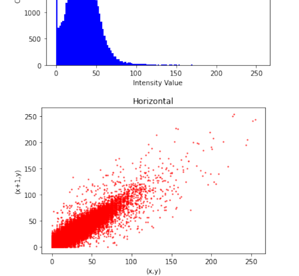

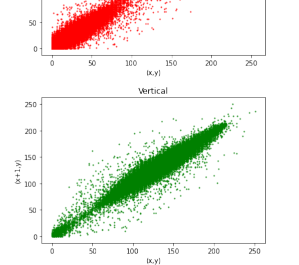

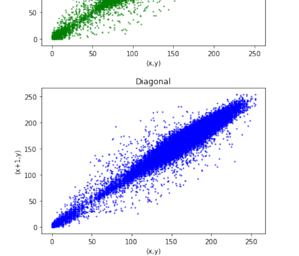

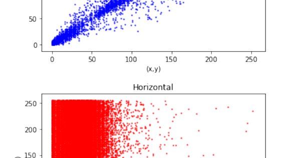

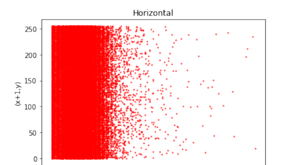

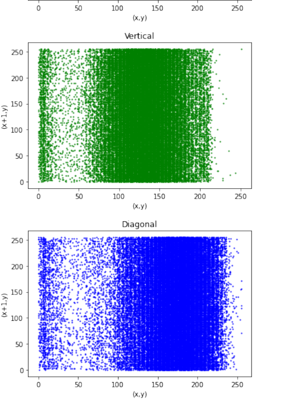

In [8]:

```
imA = io.imread("~/Desktop/Paper_Files/Images_Analysis/img/Chameleon.png")
imB = io.imread("~/Desktop/Paper_Files/Images_Analysis/img/Chameleon_E.png")

im1 = Image.open("/Users/banan/Desktop/Paper_Files/Images_Analysis/img/Chameleon
im2 = Image.open("/Users/banan/Desktop/Paper_Files/Images_Analysis/img/Chameleon

img1 = cv2.imread("/Users/banan/Desktop/Paper_Files/Images_Analysis/img/Chameleo
img2 = cv2.imread("/Users/banan/Desktop/Paper_Files/Images_Analysis/img/Chameleo
img3 = cv2.imread("/Users/banan/Desktop/Paper_Files/Images_Analysis/img/Chameleo



(scorer,scoreg,scoreb) = metrics.structural_similarity(img1[:,:,0], img2[:,:,0],

entropy  = measure.shannon_entropy(img2)
npcr  = npcrv(im1, im2)
uaci = uaciv(im1, im2)
mser,mseg,mseb  = msecalc(img1[:,:,0], img2[:,:,0]),msecalc(img1[:,:,1], img2[:,
psnrr,psnrg,psnrb = cv2.PSNR(img1[:,:,0], img2[:,:,0]),cv2.PSNR(img1[:,:,1], img

msedr,msedg,msedb  = msecalc(img1[:,:,0], img3[:,:,0]),msecalc(img1[:,:,1], img3
iqir,iqig,iqib = uqi(img1[:,:,0],img3[:,:,0]),uqi(img1[:,:,1],img3[:,:,1]), uqi(
mdr, mdg, mdb = mdv(im1,im2)
cfr, cfg, cfb = np.corrcoef(img1[:,:,0].flat, img3[:,:,0].flat)[0][1], np.corrco
cfer, cfeg, cfeb = np.corrcoef(img1[:,:,0].flat, img2[:,:,0].flat)[0][1], np.cor


print("SSIM Red: {}".format(scorer[0])+" Green: {}".format(scoreg[0])+" Blue: {}
print("Shannon Entropy: {}".format(entropy))
print("NPCR: {}".format(npcr))
print("UACI: {}".format(uaci))
print("MSE between original and encrypted Red: {}".format(mser)+" Green: {}".for
print("MSE between original and decrypted: Red: {}".format(msedr)+" Green: {}".f
print("PSNR Red: {}".format(psnrr)+" PSNR Green: {}".format(psnrg)+" PSNR Blue:
print("MD Red: {}".format(mdr)+" MD Green: {}".format(mdg)+" MD Blue: {}".format
print("IQI Red: {}".format(iqir)+" Green: {}".format(iqig)+" Blue: {}".format(iq
print("CF between original and encrypted Red: {}".format(cfer)+" Green: {}".form

_ = plt.hist(imA[:, :, 0].ravel(), bins = 128, color = 'red', alpha = 1)
_ = plt.xlabel('Intensity Value')
_ = plt.ylabel('Count')
_ = plt.legend(['Red_Channel'])
_ = plt.title('Original Chameleon')

plt.show()

_ = plt.hist(imA[:, :, 1].ravel(), bins = 128, color = 'Green', alpha = 1)
_ = plt.xlabel('Intensity Value')
_ = plt.ylabel('Count')
_ = plt.legend(['Green_Channel'])
_ = plt.title('Original Chameleon')

plt.show()

_ = plt.hist(imA[:, :, 2].ravel(), bins = 128, color = 'blue', alpha = 1)
_ = plt.xlabel('Intensity Value')
_ = plt.ylabel('Count')
_ = plt.legend(['Blue_Channel'])
_ = plt.title('Original Chameleon')

plt.show()

_ = plt.hist(imB[:, :, 0].ravel(), bins = 128, color = 'red', alpha = 1)
_ = plt.xlabel('Intensity Value')
_ = plt.ylabel('Count')
_ = plt.legend(['Red_Channel'])
_ = plt.title('Encrypted Chameleon')

plt.show()

_ = plt.hist(imB[:, :, 1].ravel(), bins = 128, color = 'Green', alpha = 1)
_ = plt.xlabel('Intensity Value')
_ = plt.ylabel('Count')
_ = plt.legend(['Green_Channel'])
_ = plt.title('Encrypted Chameleon')

plt.show()

_ = plt.hist(imB[:, :, 2].ravel(), bins = 128, color = 'blue', alpha = 1)
_ = plt.xlabel('Intensity Value')
_ = plt.ylabel('Count')
_ = plt.legend(['Blue_Channel'])
_ = plt.title('Encrypted Chameleon')

plt.show()

imC = io.imread("~/Desktop/Paper_Files/Images_Analysis/img/Chameleon_D.png")

_ = plt.hist(imC[:, :, 0].ravel(), bins = 128, color = 'red', alpha = 1)
_ = plt.xlabel('Intensity Value')
_ = plt.ylabel('Count')
_ = plt.legend(['Red_Channel'])
_ = plt.title('Decrypted Chameleon')

plt.show()

_ = plt.hist(imC[:, :, 1].ravel(), bins = 128, color = 'Green', alpha = 1)
_ = plt.xlabel('Intensity Value')
_ = plt.ylabel('Count')
_ = plt.legend(['Green_Channel'])
_ = plt.title('Decrypted Chameleon')

plt.show()

_ = plt.hist(imC[:, :, 2].ravel(), bins = 128, color = 'blue', alpha = 1)
_ = plt.xlabel('Intensity Value')
_ = plt.ylabel('Count')
_ = plt.legend(['Blue_Channel'])
_ = plt.title('Decrypted Chameleon')

plt.show()

width, height = im1.size
_ = plt.scatter(img1[1:, :, 0].ravel(),img3[:height-1, :, 0].ravel(), s=1, c = '
_ = plt.xlabel('(x,y)')
_ = plt.ylabel('(x+1,y)')
_ = plt.title('Horizontal')

plt.show()

_ = plt.scatter(img1[1:, :, 1].ravel(),img3[:height-1, :, 1].ravel(), s=1, c = '
_ = plt.xlabel('(x,y)')
_ = plt.ylabel('(x+1,y)')
_ = plt.title('Vertical')
plt.show()

_ = plt.scatter(img1[1:, :, 2].ravel(),img3[:height-1, :, 2].ravel(), s=1, c = '
_ = plt.xlabel('(x,y)')
_ = plt.ylabel('(x+1,y)')
_ = plt.title('Diagonal')
plt.show()

_ = plt.scatter(img1[1:, :, 0].ravel(),img2[:height-1, :, 0].ravel(), s=1, c = '
_ = plt.xlabel('(x,y)')
_ = plt.ylabel('(x+1,y)')
_ = plt.title('Horizontal')
plt.show()

_ = plt.scatter(img1[1:, :, 1].ravel(),img2[:height-1, :, 1].ravel(), s=1, c = '
_ = plt.xlabel('(x,y)')
_ = plt.ylabel('(x+1,y)')
_ = plt.title('Vertical')
plt.show()

_ = plt.scatter(img1[1:, :, 2].ravel(),img2[:height-1, :, 2].ravel(), s=1, c = '
_ = plt.xlabel('(x,y)')
_ = plt.ylabel('(x+1,y)')
_ = plt.title('Diagonal')
plt.show()
```

```
SSIM Red: 0.004708305993219484 Green: 0.008266583804995469 Blue: 0.0079904128005
2114
Shannon Entropy: 7.989400594572217
NPCR: 99.65336595771379
UACI: (37.082537304199825, 34.94748360478246, 33.31321988612096, 33.313219886120
96)
MSE between original and encrypted Red: 17154.834070518853 Green: 10852.10035133
9483 Blue: 11914.7619518163
MSE between original and decrypted: Red: 0.2588148566409436 Green: 0.65603237342
36778 Blue: 0.5417686178555744
PSNR Red: 5.78693839152288 PSNR Green: 7.775665597599698 PSNR Blue: 7.3699499098
31996
MD Red: 255 MD Green: 255 MD Blue: 255
IQI Red: 0.9999948175657052 Green: 0.9999799170682709 Blue: 0.9999863736573865
CF between original and encrypted Red: 0.002088302016329925 Green: -0.0010186158
019077141 Blue: -0.003147461072935109
 CF btween original and decrypted Red: 0.9999991657813831 Green: 0.9999886776106
632 Blue: 0.9999944425482403
```

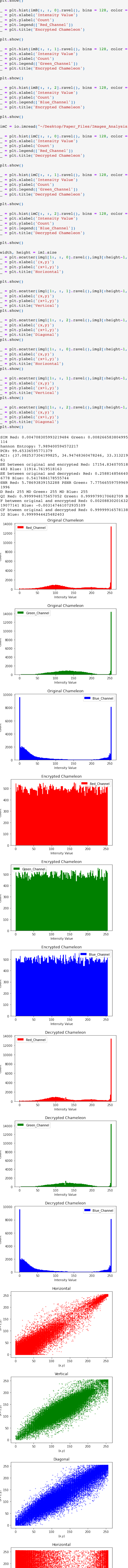

In [9]:

```
imA = io.imread("~/Desktop/Paper_Files/Images_Analysis/img/Pepper.png")
imB = io.imread("~/Desktop/Paper_Files/Images_Analysis/img/Pepper_E.png")

im1 = Image.open("/Users/banan/Desktop/Paper_Files/Images_Analysis/img/Pepper.pn
im2 = Image.open("/Users/banan/Desktop/Paper_Files/Images_Analysis/img/Pepper_E.

img1 = cv2.imread("/Users/banan/Desktop/Paper_Files/Images_Analysis/img/Pepper.p
img2 = cv2.imread("/Users/banan/Desktop/Paper_Files/Images_Analysis/img/Pepper_E
img3 = cv2.imread("/Users/banan/Desktop/Paper_Files/Images_Analysis/img/Pepper_D



(scorer,scoreg,scoreb) = metrics.structural_similarity(img1[:,:,0], img2[:,:,0],

entropy  = measure.shannon_entropy(img2)
npcr  = npcrv(im1, im2)
uaci = uaciv(im1, im2)
mser,mseg,mseb  = msecalc(img1[:,:,0], img2[:,:,0]),msecalc(img1[:,:,1], img2[:,
psnrr,psnrg,psnrb = cv2.PSNR(img1[:,:,0], img2[:,:,0]),cv2.PSNR(img1[:,:,1], img

msedr,msedg,msedb  = msecalc(img1[:,:,0], img3[:,:,0]),msecalc(img1[:,:,1], img3
iqir,iqig,iqib = uqi(img1[:,:,0],img3[:,:,0]),uqi(img1[:,:,1],img3[:,:,1]), uqi(
mdr, mdg, mdb = mdv(im1,im2)
cfr, cfg, cfb = np.corrcoef(img1[:,:,0].flat, img3[:,:,0].flat)[0][1], np.corrco
cfer, cfeg, cfeb = np.corrcoef(img1[:,:,0].flat, img2[:,:,0].flat)[0][1], np.cor


print("SSIM Red: {}".format(scorer[0])+" Green: {}".format(scoreg[0])+" Blue: {}
print("Shannon Entropy: {}".format(entropy))
print("NPCR: {}".format(npcr))
print("UACI: {}".format(uaci))
print("MSE between original and encrypted Red: {}".format(mser)+" Green: {}".for
print("MSE between original and decrypted: Red: {}".format(msedr)+" Green: {}".f
print("PSNR Red: {}".format(psnrr)+" PSNR Green: {}".format(psnrg)+" PSNR Blue:
print("MD Red: {}".format(mdr)+" MD Green: {}".format(mdg)+" MD Blue: {}".format
print("IQI Red: {}".format(iqir)+" Green: {}".format(iqig)+" Blue: {}".format(iq
print("CF between original and encrypted Red: {}".format(cfer)+" Green: {}".form

_ = plt.hist(imA[:, :, 0].ravel(), bins = 128, color = 'red', alpha = 1)
_ = plt.xlabel('Intensity Value')
_ = plt.ylabel('Count')
_ = plt.legend(['Red_Channel'])
_ = plt.title('Original Pepper')

plt.show()

_ = plt.hist(imA[:, :, 1].ravel(), bins = 128, color = 'Green', alpha = 1)
_ = plt.xlabel('Intensity Value')
_ = plt.ylabel('Count')
_ = plt.legend(['Green_Channel'])
_ = plt.title('Original Pepper')

plt.show()

_ = plt.hist(imA[:, :, 2].ravel(), bins = 128, color = 'blue', alpha = 1)
_ = plt.xlabel('Intensity Value')
_ = plt.ylabel('Count')
_ = plt.legend(['Blue_Channel'])
_ = plt.title('Original Pepper')

plt.show()

_ = plt.hist(imB[:, :, 0].ravel(), bins = 128, color = 'red', alpha = 1)
_ = plt.xlabel('Intensity Value')
_ = plt.ylabel('Count')
_ = plt.legend(['Red_Channel'])
_ = plt.title('Encrypted Pepper')

plt.show()

_ = plt.hist(imB[:, :, 1].ravel(), bins = 128, color = 'Green', alpha = 1)
_ = plt.xlabel('Intensity Value')
_ = plt.ylabel('Count')
_ = plt.legend(['Green_Channel'])
_ = plt.title('Encrypted Pepper')

plt.show()

_ = plt.hist(imB[:, :, 2].ravel(), bins = 128, color = 'blue', alpha = 1)
_ = plt.xlabel('Intensity Value')
_ = plt.ylabel('Count')
_ = plt.legend(['Blue_Channel'])
_ = plt.title('Encrypted Pepper')

plt.show()

imC = io.imread("~/Desktop/Paper_Files/Images_Analysis/img/Pepper_D.png")

_ = plt.hist(imC[:, :, 0].ravel(), bins = 128, color = 'red', alpha = 1)
_ = plt.xlabel('Intensity Value')
_ = plt.ylabel('Count')
_ = plt.legend(['Red_Channel'])
_ = plt.title('Decrypted Pepper')

plt.show()

_ = plt.hist(imC[:, :, 1].ravel(), bins = 128, color = 'Green', alpha = 1)
_ = plt.xlabel('Intensity Value')
_ = plt.ylabel('Count')
_ = plt.legend(['Green_Channel'])
_ = plt.title('Decrypted Pepper')

plt.show()

_ = plt.hist(imC[:, :, 2].ravel(), bins = 128, color = 'blue', alpha = 1)
_ = plt.xlabel('Intensity Value')
_ = plt.ylabel('Count')
_ = plt.legend(['Blue_Channel'])
_ = plt.title('Decrypted Pepper')

plt.show()

width, height = im1.size
_ = plt.scatter(img1[1:, :, 0].ravel(),img3[:height-1, :, 0].ravel(), s=1, c = '
_ = plt.xlabel('(x,y)')
_ = plt.ylabel('(x+1,y)')
_ = plt.title('Horizontal')

plt.show()

_ = plt.scatter(img1[1:, :, 1].ravel(),img3[:height-1, :, 1].ravel(), s=1, c = '
_ = plt.xlabel('(x,y)')
_ = plt.ylabel('(x+1,y)')
[illegible]
```

[illegible]

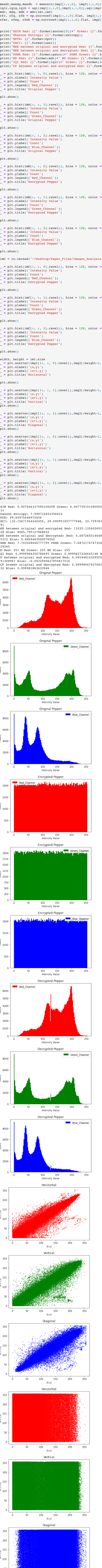

In [10]:
```
imA = io.imread("~/Desktop/Paper_Files/Images_Analysis/img/Tree.png")
imB = io.imread("~/Desktop/Paper_Files/Images_Analysis/img/Tree_E.png")

im1 = Image.open("/Users/banan/Desktop/Paper_Files/Images_Analysis/img/Tree.png"
im2 = Image.open("/Users/banan/Desktop/Paper_Files/Images_Analysis/img/Tree_E.pn

img1 = cv2.imread("/Users/banan/Desktop/Paper_Files/Images_Analysis/img/Tree.png
img2 = cv2.imread("/Users/banan/Desktop/Paper_Files/Images_Analysis/img/Tree_E.p
img3 = cv2.imread("/Users/banan/Desktop/Paper_Files/Images_Analysis/img/Tree_D.p



(scorer,scoreg,scoreb) = metrics.structural_similarity(img1[:,:,0], img2[:,:,0],

entropy  = measure.shannon_entropy(img2)
npcr  = npcrv(im1, im2)
uaci = uaciv(im1, im2)
mser,mseg,mseb  = msecalc(img1[:,:,0], img2[:,:,0]),msecalc(img1[:,:,1], img2[:,
psnrr,psnrg,psnrb = cv2.PSNR(img1[:,:,0], img2[:,:,0]),cv2.PSNR(img1[:,:,1], img

msedr,msedg,msedb  = msecalc(img1[:,:,0], img3[:,:,0]),msecalc(img1[:,:,1], img3
iqir,iqig,iqib = uqi(img1[:,:,0],img3[:,:,0]),uqi(img1[:,:,1],img3[:,:,1]), uqi(
mdr, mdg, mdb = mdv(im1,im2)
cfr, cfg, cfb = np.corrcoef(img1[:,:,0].flat, img3[:,:,0].flat)[0][1], np.corrco
cfer, cfeg, cfeb = np.corrcoef(img1[:,:,0].flat, img2[:,:,0].flat)[0][1], np.cor


print("SSIM Red: {}".format(scorer[0])+" Green: {}".format(scoreg[0])+" Blue: {}
print("Shannon Entropy: {}".format(entropy))
print("NPCR: {}".format(npcr))
print("UACI: {}".format(uaci))
print("MSE between original and encrypted Red: {}".format(mser)+" Green: {}".for
print("MSE between original and decrypted: Red: {}".format(msedr)+" Green: {}".f
print("PSNR Red: {}".format(psnrr)+" PSNR Green: {}".format(psnrg)+" PSNR Blue:
print("MD Red: {}".format(mdr)+" MD Green: {}".format(mdg)+" MD Blue: {}".format
print("IQI Red: {}".format(iqir)+" Green: {}".format(iqig)+" Blue: {}".format(iq
print("CF between original and encrypted Red: {}".format(cfer)+" Green: {}".form

_ = plt.hist(imA[:, :, 0].ravel(), bins = 128, color = 'red', alpha = 1)
_ = plt.xlabel('Intensity Value')
_ = plt.ylabel('Count')
_ = plt.legend(['Red_Channel'])
_ = plt.title('Original Tree')

plt.show()

_ = plt.hist(imA[:, :, 1].ravel(), bins = 128, color = 'Green', alpha = 1)
_ = plt.xlabel('Intensity Value')
_ = plt.ylabel('Count')
_ = plt.legend(['Green_Channel'])
_ = plt.title('Original Tree')

plt.show()

_ = plt.hist(imA[:, :, 2].ravel(), bins = 128, color = 'blue', alpha = 1)
_ = plt.xlabel('Intensity Value')
_ = plt.ylabel('Count')
_ = plt.legend(['Blue_Channel'])
_ = plt.title('Original Tree')

plt.show()

_ = plt.hist(imB[:, :, 0].ravel(), bins = 128, color = 'red', alpha = 1)
_ = plt.xlabel('Intensity Value')
_ = plt.ylabel('Count')
_ = plt.legend(['Red_Channel'])
_ = plt.title('Encrypted Tree')

plt.show()

_ = plt.hist(imB[:, :, 1].ravel(), bins = 128, color = 'Green', alpha = 1)
_ = plt.xlabel('Intensity Value')
_ = plt.ylabel('Count')
_ = plt.legend(['Green_Channel'])
_ = plt.title('Encrypted Tree')

plt.show()

_ = plt.hist(imB[:, :, 2].ravel(), bins = 128, color = 'blue', alpha = 1)
_ = plt.xlabel('Intensity Value')
_ = plt.ylabel('Count')
_ = plt.legend(['Blue_Channel'])
_ = plt.title('Encrypted Tree')

plt.show()

imC = io.imread("~/Desktop/Paper_Files/Images_Analysis/img/Tree_D.png")

_ = plt.hist(imC[:, :, 0].ravel(), bins = 128, color = 'red', alpha = 1)
_ = plt.xlabel('Intensity Value')
_ = plt.ylabel('Count')
_ = plt.legend(['Red_Channel'])
_ = plt.title('Decrypted Tree')

plt.show()

_ = plt.hist(imC[:, :, 1].ravel(), bins = 128, color = 'Green', alpha = 1)
_ = plt.xlabel('Intensity Value')
_ = plt.ylabel('Count')
_ = plt.legend(['Green_Channel'])
_ = plt.title('Decrypted Tree')

plt.show()

_ = plt.hist(imC[:, :, 2].ravel(), bins = 128, color = 'blue', alpha = 1)
_ = plt.xlabel('Intensity Value')
_ = plt.ylabel('Count')
_ = plt.legend(['Blue_Channel'])
_ = plt.title('Decrypted Tree')

plt.show()

width, height = im1.size
_ = plt.scatter(img1[1:, :, 0].ravel(),img3[:height-1, :, 0].ravel(), s=1, c = '
_ = plt.xlabel('(x,y)')
_ = plt.ylabel('(x+1,y)')
_ = plt.title('Horizontal')

plt.show()

_ = plt.scatter(img1[1:, :, 1].ravel(),img3[:height-1, :, 1].ravel(), s=1, c = '
_ = plt.xlabel('(x,y)')
_ = plt.ylabel('(x+1,y)')
_ = plt.title('Vertical')
plt.show()

_ = plt.scatter(img1[1:, :, 2].ravel(),img3[:height-1, :, 2].ravel(), s=1, c = '
_ = plt.xlabel('(x,y)')
_ = plt.ylabel('(x+1,y)')
_ = plt.title('Diagonal')
plt.show()

_ = plt.scatter(img1[1:, :, 0].ravel(),img2[:height-1, :, 0].ravel(), s=1, c = '
_ = plt.xlabel('(x,y)')
_ = plt.ylabel('(x+1,y)')
_ = plt.title('Horizontal')
plt.show()

_ = plt.scatter(img1[1:, :, 1].ravel(),img2[:height-1, :, 1].ravel(), s=1, c = '
_ = plt.xlabel('(x,y)')
_ = plt.ylabel('(x+1,y)')
_ = plt.title('Vertical')
plt.show()

_ = plt.scatter(img1[1:, :, 2].ravel(),img2[:height-1, :, 2].ravel(), s=1, c = '
_ = plt.xlabel('(x,y)')
_ = plt.ylabel('(x+1,y)')
_ = plt.title('Diagonal')
plt.show()
```

```
SSIM Red: 0.004933450582316831 Green: 0.008277377448449728 Blue: 0.0117577954963
47959
Shannon Entropy: 7.998419532631743
NPCR: 99.61083333333333
UACI: (30.697133986931195, 29.99702941176689, 30.183892156865454, 30.18389215686
5454)
MSE between original and encrypted Red: 9908.858725 Green: 8806.393275 Blue: 869
8.995025
MSE between original and decrypted: Red: 0.8514 Green: 0.856125 Blue: 0.862375
PSNR Red: 8.1705672434202 PSNR Green: 8.682822846332755 PSNR Blue: 8.73611278399
5571
MD Red: 237 MD Green: 237 MD Blue: 235
IQI Red: 0.9999741931951677 Green: 0.9999726316457591 Blue: 0.999971852403414
CF between original and encrypted Red: -0.01045025149702007 Green: 0.00544524553
9735738 Blue: 0.004829078785955487
 CF btween original and decrypted Red: 0.9999880270020608 Green: 0.9999853556477
577 Blue: 0.9999841498597196
```

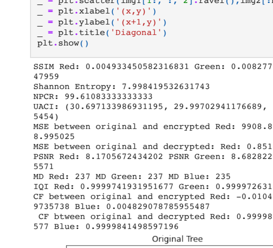

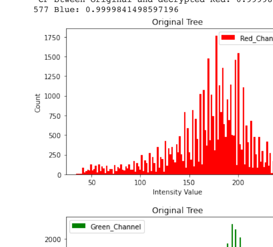

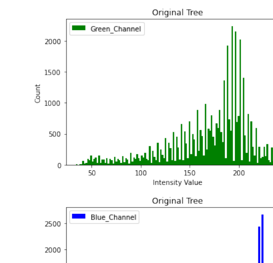

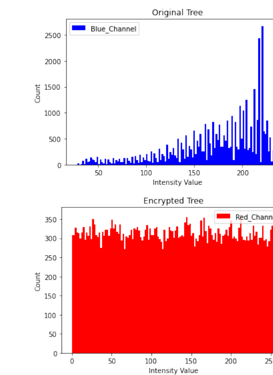

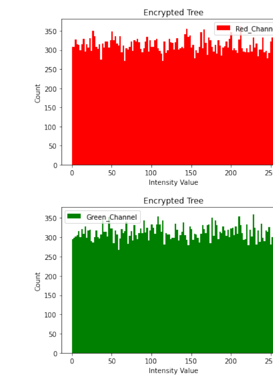

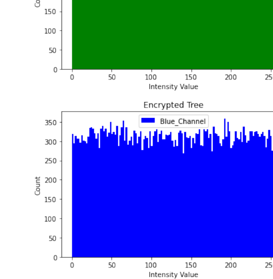

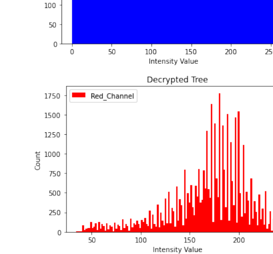

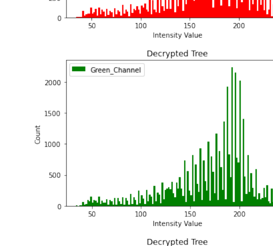

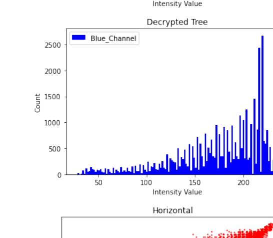

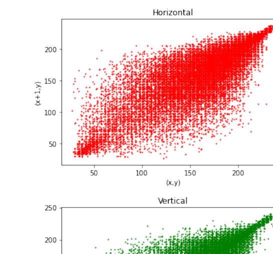

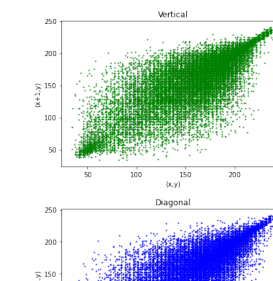

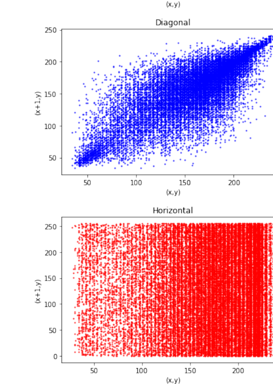

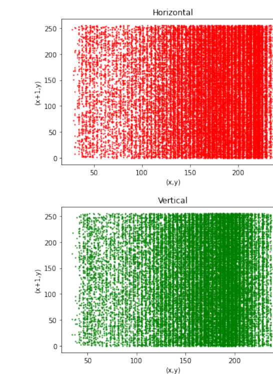

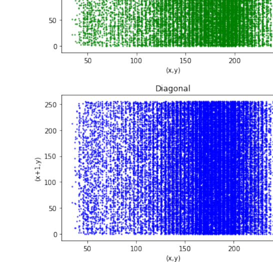

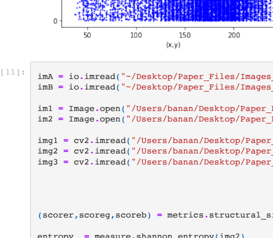

In [11]:
```
imA = io.imread("~/Desktop/Paper_Files/Images_Analysis/img/Lighthouse.png")
imB = io.imread("~/Desktop/Paper_Files/Images_Analysis/img/Lighthouse_E.png")

im1 = Image.open("/Users/banan/Desktop/Paper_Files/Images_Analysis/img/Lighthous
im2 = Image.open("/Users/banan/Desktop/Paper_Files/Images_Analysis/img/Lighthous

img1 = cv2.imread("/Users/banan/Desktop/Paper_Files/Images_Analysis/img/Lighthou
img2 = cv2.imread("/Users/banan/Desktop/Paper_Files/Images_Analysis/img/Lighthou
img3 = cv2.imread("/Users/banan/Desktop/Paper_Files/Images_Analysis/img/Lighthou



(scorer,scoreg,scoreb) = metrics.structural_similarity(img1[:,:,0], img2[:,:,0],

entropy  = measure.shannon_entropy(img2)
npcr  = npcrv(im1, im2)
uaci = uaciv(im1, im2)
mser,mseg,mseb  = msecalc(img1[:,:,0], img2[:,:,0]),msecalc(img1[:,:,1], img2[:,
psnrr,psnrg,psnrb = cv2.PSNR(img1[:,:,0], img2[:,:,0]),cv2.PSNR(img1[:,:,1], img

msedr,msedg,msedb  = msecalc(img1[:,:,0], img3[:,:,0]),msecalc(img1[:,:,1], img3
iqir,iqig,iqib = uqi(img1[:,:,0],img3[:,:,0]),uqi(img1[:,:,1],img3[:,:,1]), uqi(
mdr, mdg, mdb = mdv(im1,im2)
cfr, cfg, cfb = np.corrcoef(img1[:,:,0].flat, img3[:,:,0].flat)[0][1], np.corrco
cfer, cfeg, cfeb = np.corrcoef(img1[:,:,0].flat, img2[:,:,0].flat)[0][1], np.cor


print("SSIM Red: {}".format(scorer[0])+" Green: {}".format(scoreg[0])+" Blue: {}
print("Shannon Entropy: {}".format(entropy))
print("NPCR: {}".format(npcr))
print("UACI: {}".format(uaci))
print("MSE between original and encrypted Red: {}".format(mser)+" Green: {}".for
print("MSE between original and decrypted: Red: {}".format(msedr)+" Green: {}".f
print("PSNR Red: {}".format(psnrr)+" PSNR Green: {}".format(psnrg)+" PSNR Blue:
print("MD Red: {}".format(mdr)+" MD Green: {}".format(mdg)+" MD Blue: {}".format
print("IQI Red: {}".format(iqir)+" Green: {}".format(iqig)+" Blue: {}".format(iq
print("CF between original and encrypted Red: {}".format(cfer)+" Green: {}".form

_ = plt.hist(imA[:, :, 0].ravel(), bins = 128, color = 'red', alpha = 1)
_ = plt.xlabel('Intensity Value')
_ = plt.ylabel('Count')
_ = plt.legend(['Red_Channel'])
_ = plt.title('Original Lighthouse')

plt.show()

_ = plt.hist(imA[:, :, 1].ravel(), bins = 128, color = 'Green', alpha = 1)
_ = plt.xlabel('Intensity Value')
_ = plt.ylabel('Count')
_ = plt.legend(['Green_Channel'])
_ = plt.title('Original Lighthouse')

plt.show()

_ = plt.hist(imA[:, :, 2].ravel(), bins = 128, color = 'blue', alpha = 1)
_ = plt.xlabel('Intensity Value')
_ = plt.ylabel('Count')
_ = plt.legend(['Blue_Channel'])
_ = plt.title('Original Lighthouse')

plt.show()

_ = plt.hist(imB[:, :, 0].ravel(), bins = 128, color = 'red', alpha = 1)
_ = plt.xlabel('Intensity Value')
_ = plt.ylabel('Count')
_ = plt.legend(['Red_Channel'])
_ = plt.title('Encrypted Lighthouse')

plt.show()

_ = plt.hist(imB[:, :, 1].ravel(), bins = 128, color = 'Green', alpha = 1)
_ = plt.xlabel('Intensity Value')
_ = plt.ylabel('Count')
_ = plt.legend(['Green_Channel'])
_ = plt.title('Encrypted Lighthouse')

plt.show()

_ = plt.hist(imB[:, :, 2].ravel(), bins = 128, color = 'blue', alpha = 1)
_ = plt.xlabel('Intensity Value')
_ = plt.ylabel('Count')
_ = plt.legend(['Blue_Channel'])
_ = plt.title('Encrypted Lighthouse')

plt.show()

imC = io.imread("~/Desktop/Paper_Files/Images_Analysis/img/Lighthouse_D.png")

_ = plt.hist(imC[:, :, 0].ravel(), bins = 128, color = 'red', alpha = 1)
_ = plt.xlabel('Intensity Value')
_ = plt.ylabel('Count')
_ = plt.legend(['Red_Channel'])
_ = plt.title('Decrypted Lighthouse')

plt.show()

_ = plt.hist(imC[:, :, 1].ravel(), bins = 128, color = 'Green', alpha = 1)
_ = plt.xlabel('Intensity Value')
_ = plt.ylabel('Count')
_ = plt.legend(['Green_Channel'])
_ = plt.title('Decrypted Lighthouse')

plt.show()

_ = plt.hist(imC[:, :, 2].ravel(), bins = 128, color = 'blue', alpha = 1)
_ = plt.xlabel('Intensity Value')
_ = plt.ylabel('Count')
_ = plt.legend(['Blue_Channel'])
_ = plt.title('Decrypted Lighthouse')

plt.show()

width, height = im1.size
_ = plt.scatter(img1[1:, :, 0].ravel(),img3[:height-1, :, 0].ravel(), s=1, c = '
_ = plt.xlabel('(x,y)')
_ = plt.ylabel('(x+1,y)')
_ = plt.title('Horizontal')

plt.show()

_ = plt.scatter(img1[1:, :, 1].ravel(),img3[:height-1, :, 1].ravel(), s=1, c = '
_ = plt.xlabel('(x,y)')
_ = plt.ylabel('(x+1,y)')
_ = plt.title('Vertical')
plt.show()

_ = plt.scatter(img1[1:, :, 2].ravel(),img3[:height-1, :, 2].ravel(), s=1, c = '
_ = plt.xlabel('(x,y)')
_ = plt.ylabel('(x+1,y)')
_ = plt.title('Diagonal')
plt.show()

_ = plt.scatter(img1[1:, :, 0].ravel(),img2[:height-1, :, 0].ravel(), s=1, c = '
_ = plt.xlabel('(x,y)')
_ = plt.ylabel('(x+1,y)')
_ = plt.title('Horizontal')
plt.show()

_ = plt.scatter(img1[1:, :, 1].ravel(),img2[:height-1, :, 1].ravel(), s=1, c = '
_ = plt.xlabel('(x,y)')
_ = plt.ylabel('(x+1,y)')
_ = plt.title('Vertical')
plt.show()

_ = plt.scatter(img1[1:, :, 2].ravel(),img2[:height-1, :, 2].ravel(), s=1, c = '
_ = plt.xlabel('(x,y)')
_ = plt.ylabel('(x+1,y)')
_ = plt.title('Diagonal')
plt.show()
```

```
SSIM Red: 0.012235306503510951 Green: 0.011026863890624622 Blue: 0.0073961932393
559904
Shannon Entropy: 7.999253319180162
NPCR: 99.60295002739825
UACI: (29.044187882038738, 29.039053463309212, 28.52405629296404, 28.52405629296
404)
MSE between original and encrypted Red: 8374.803002128978 Green: 7683.1357291077
16 Blue: 8024.709165386585
MSE between original and decrypted: Red: 0.2712426226857466 Green: 0.34780499636
18724 Blue: 0.37321799121459565
PSNR Red: 8.9010576081211 PSNR Green: 9.275418554317586 PSNR Blue: 9.08651059374
3744
MD Red: 255 MD Green: 251 MD Blue: 255
IQI Red: 0.9999870177560829 Green: 0.9999862505542755 Blue: 0.999986584406657
CF between original and encrypted Red: 0.009881891936673179 Green: -0.0006915344
184191888 Blue: -0.006863509325753056
 CF btween original and decrypted Red: 0.9999731659798328 Green: 0.9999805724166
737 Blue: 0.9999844427069604
```

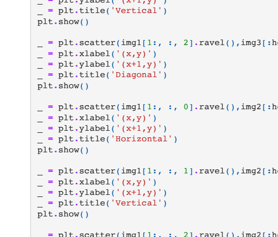

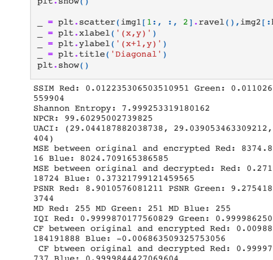

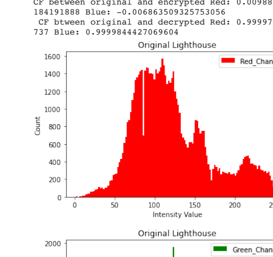

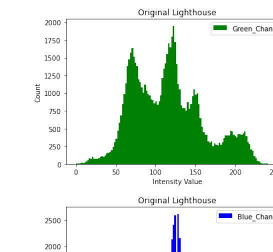

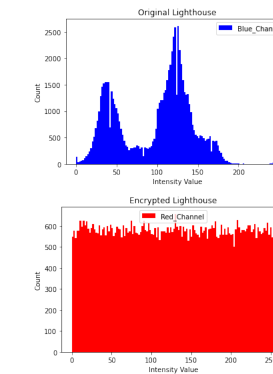

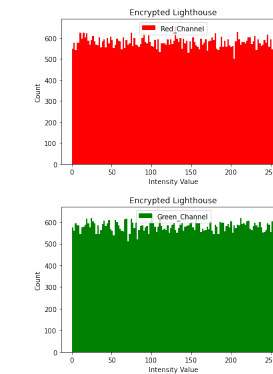

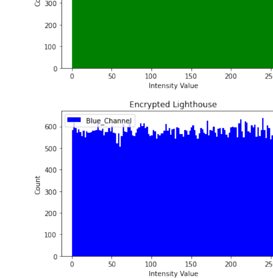

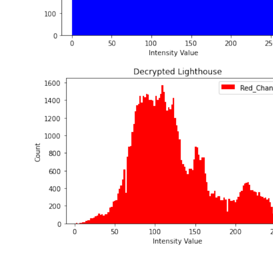

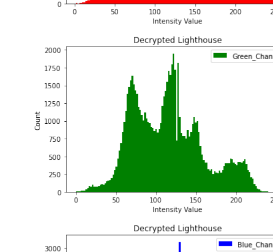

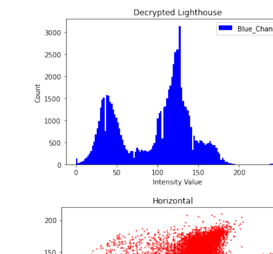

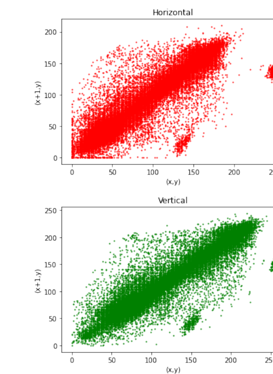

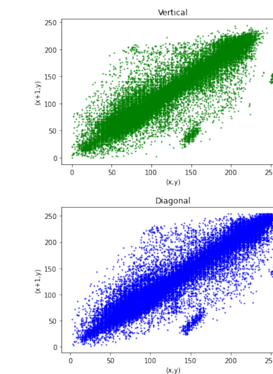

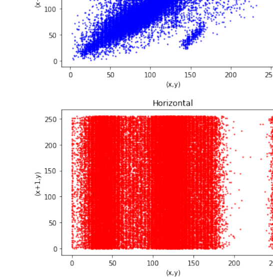

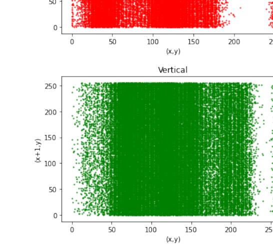

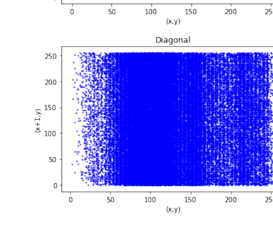

Supplement: Supplemental Information 1 — Includes the numerical and graphical results. [file peerj-cs-07-812-s001.pdf]
